# A plant DNA virus replicates in the salivary glands of its insect vector via recruitment of host DNA synthesis machinery

**DOI:** 10.1073/pnas.1820132117

**Published:** 2020-07-07

**Authors:** Ya-Zhou He, Yu-Meng Wang, Tian-Yan Yin, Elvira Fiallo-Olivé, Yin-Quan Liu, Linda Hanley-Bowdoin, Xiao-Wei Wang

**Affiliations:** ^a^Ministry of Agriculture Key Laboratory of Molecular Biology of Crop Pathogens and Insects, Institute of Insect Sciences, Zhejiang University, 310058 Hangzhou, China;; ^b^Instituto de Hortofruticultura Subtropical y Mediterránea La Mayora, Universidad de Málaga–Consejo Superior de Investigaciones Científicas, Estación Experimental La Mayora, 29750 Algarrobo-Costa, Málaga, Spain;; ^c^Department of Plant and Microbial Biology, North Carolina State University, Raleigh, NC 27695-7651

**Keywords:** DNA synthesis machinery, insect vector, plant DNA virus, replication, salivary glands

## Abstract

Whereas most of the arthropod-borne animal viruses replicate in their vectors, this is less common for plant viruses. So far, only some plant RNA viruses have been demonstrated to replicate in insect vectors and plant hosts. How plant viruses evolved to replicate in the animal kingdom remains largely unknown. Geminiviruses comprise a large family of plant-infecting, single-stranded DNA viruses that cause serious crop losses worldwide. Here, we report evidence and insight into the replication of the geminivirus tomato yellow leaf curl virus (TYLCV) in the whitefly (*Bemisia tabaci*) vector and that replication is mainly in the salivary glands. We found that TYLCV induces DNA synthesis machinery, proliferating cell nuclear antigen (PCNA) and DNA polymerase δ (Polδ), to establish a replication-competent environment in whiteflies. TYLCV replication-associated protein (Rep) interacts with whitefly PCNA, which recruits DNA Polδ for virus replication. In contrast, another geminivirus, papaya leaf curl China virus (PaLCuCNV), does not replicate in the whitefly vector. PaLCuCNV does not induce DNA-synthesis machinery, and the Rep does not interact with whitefly PCNA. Our findings reveal important mechanisms by which a plant DNA virus replicates across the kingdom barrier in an insect and may help to explain the global spread of this devastating pathogen.

## Significance

Viruses pose a great threat to animal and plant health worldwide. Whereas most plant viruses only replicate in plant hosts, some also replicate in their animal (insect) vector. A detailed knowledge of host expansion will give a better understanding of virus evolution, and identification of virus and host components involved in this process can lead to new strategies to combat virus spread. Here, we reveal that a plant DNA virus has evolved to induce and recruit insect DNA synthesis machinery to support its replication in vector salivary glands. Our study sheds light on the understanding of TYLCV–whitefly interactions and provides insights into how a plant virus may evolve to infect and replicate in an insect vector.

Many viruses, including those that cause significant global problems in humans, animals, and plants, rely on arthropod vectors to move from one host to another (1, 2). Whereas most of the arthropod-borne animal viruses are able to replicate in their vectors, replication within the insect vector is far less common for plant viruses because of the different physiological properties between the plant and animal kingdoms (1, 3). Of the ∼1,100 known plant viruses, more than 75% are transmitted by aphids, whiteflies, leafhoppers, planthoppers, and other insects in a nonpersistent, semipersistent, or persistent manner (3, 4). Viruses in the first two categories are retained by the vector at the stylet or foregut and can only be transmitted from a few hours to a few days after acquisition (4). In contrast, persistent viruses move through the insect body. Some replicate in organs of the vector and are classified as persistent-propagative viruses, whereas the others that do not replicate are classified as persistent-circulative viruses (3). Upon acquisition, persistent-propagative viruses can be transmitted to plants for the life of the insect and are often transmitted transovarially to the vector’s progeny population (3, 5, 6). So far, only some plant RNA viruses—i.e., reoviruses, rhabdoviruses, tospoviruses, marafiviruses, and tenuiviruses—have been demonstrated to replicate within their insect vectors in addition to their plant hosts (3). Whether plant DNA viruses have also evolved to replicate in their insect vectors is not clearly known. More importantly, the mechanisms used by plant viruses to cross the kingdom barrier to replicate within insect vectors have rarely been reported. This question is of major importance because knowledge of host expansion will give a better understanding of virus evolution, and identification of virus and vector components involved in replication could lead to new strategies to interdict virus spread.

Geminiviruses are a large family of plant-infecting DNA viruses that cause serious crop losses worldwide (7). Geminivirus genomes consist of circular, single-stranded DNA (ssDNA), which is packaged into twin-shaped, quasi-icosahedral virions (8). After transmission to the plant, the viral ssDNA is released from the virion and converted to the double-stranded DNA (dsDNA) replicative form with the aid of host enzymes in the nucleus. The dsDNA is then transcribed by host RNA polymerase II, allowing the production of the replication-associated protein (Rep). This protein initiates viral replication, and complementary-sense (CS) and virion-sense (VS) DNA strands are produced by a combination of rolling-circle replication and recombination-dependent replication (9, 10). Finally, the circular, VS ssDNA is encapsulated by the viral coat protein (CP) into new viral particles. Complex interactions occur between virus-encoded proteins and host components to redirect host machineries and processes for a productive infection (11).

Begomoviruses, which constitute the largest geminivirus genus, are exclusively transmitted by the whitefly *Bemisia tabaci* cryptic species complex in a persistent-circulative manner (12, 13). These viruses circulate in the whitefly body, from the midgut (MG) lumen into the hemolymph and, finally, into the primary salivary glands (PSGs), from which they are introduced back into the plant host during insect feeding (14). During the past 30 y, with the global invasion of two species of the *B. tabaci* complex, Middle East Asia Minor 1 (MEAM1; previously biotype B) and Mediterranean (MED; previously biotype Q) begomoviruses have emerged as serious constraints to the cultivation of a variety of economically important crops worldwide (15, 16). Begomoviruses are widely believed to be unable to replicate in their insect vectors (13, 16). A notable exception is tomato yellow leaf curl virus (TYLCV), one of the most devastating viral pathogens of tomato that has spread to more than 50 countries and regions (15). The possible replication of TYLCV in the whitefly vector has received considerable attention since 1994 (17–23). Whereas several studies provided evidence supporting replication of TYLCV in *B. tabaci* (17–21), other studies have reported that the virus does not replicate in the insect vector (22, 23). Therefore, much remains to be learned about whether and how TYLCV replicates within its insect vector.

Previously, we demonstrated that TYLCV could be transmitted transovarially from viruliferous whiteflies to their progeny for at least two generations in the MEAM1 and MED cryptic species of *B. tabaci* (24), which is not typically observed for persistent-circulative transmitted viruses (1, 3). Here, we report evidence that TYLCV replicates in the whitefly vector, occurring primarily in cells of the PSGs. Moreover, we found that TYLCV induces and recruits whitefly proliferating cell nuclear antigen (PCNA) to support its replication in the vector, a mechanism that the plant DNA virus has used to replicate across the kingdom barrier.

## Results

### Dynamics of TYLCV in the Adult Offspring of Viruliferous MEAM1 Whiteflies.

**Fig. 1.**
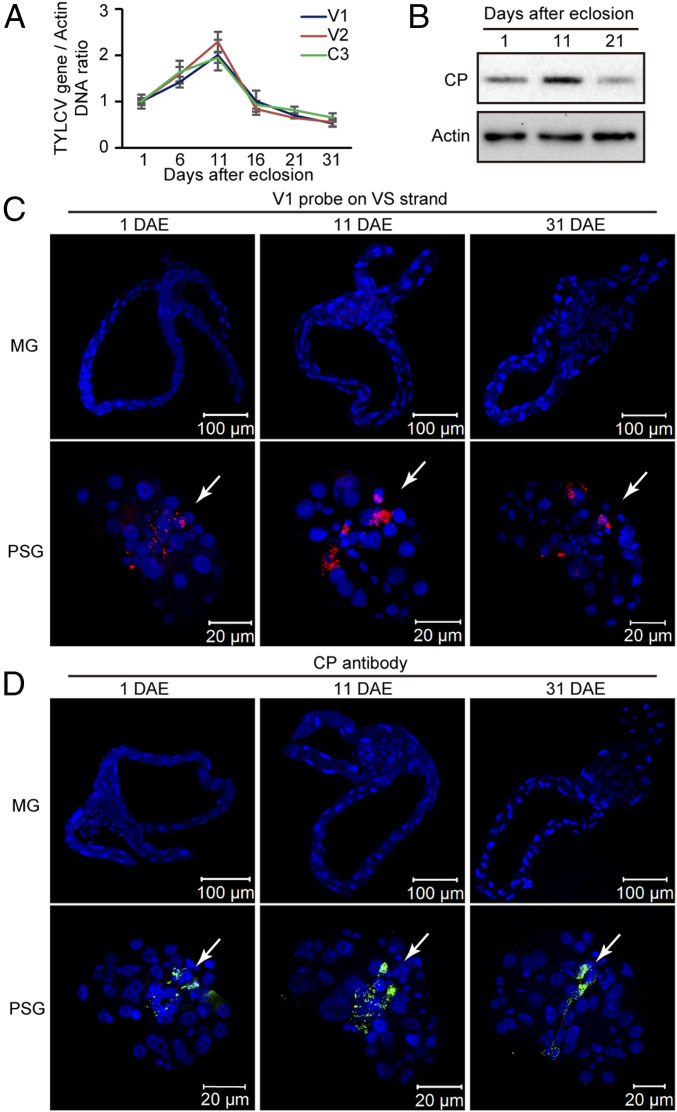
Dynamics of TYLCV in adult offspring of viruliferous MEAM1 whiteflies. (*A*) Relative concentration of TYLCV DNA in whole bodies of F1 adults at different developmental stages obtained by amplifying portions of the *V1*, *V2*, and *C3* genes using qPCR. Mean ± SEM of three independent experiments is shown. (*B*) Accumulation of TYLCV CP in F1 adults at different developmental stages. (*C* and *D*) Localization of TYLCV VS DNA (*C*) and CP (*D*) in MGs and PSGs of F1 adults at different developmental stages. For TYLCV VS DNA localization, MGs and PSGs were hybridized with a Cy3-labeled VS strand-specific probe (V1 probe; red). TYLCV CP was detected by use of a mouse anti-CP monoclonal antibody and goat anti-mouse IgG labeled with Dylight 488 (green) secondary antibody. Cell nucleus was stained with DAPI (blue). The white arrow indicates of the virus signal in the PSG. For each time point, 20 samples were analyzed, and a similar trend was observed.

Previously, we demonstrated transovarial transmission of TYLCV in MEAM1 whiteflies and that all developmental stages of their progeny accumulate the virus efficiently (68 to 92%), and that adult offspring are able to transmit TYLCV to tomato plants (24). Given that this type of transmission usually indicates that the virus is replicating in the vector, we examined the dynamics of TYLCV in whole bodies of first-generation (F1) adults, which developed from eggs deposited on cotton by viruliferous MEAM1 whiteflies. Female adult progenies were collected in groups of 10 at 1, 6, 11, 16, 21, and 31 d after eclosion (DAE) and used for viral genomic DNA abundance determination by normalized qPCR with primers specific for the *V1*, *V2*, and *C3* genes. Regardless of the primers used and the gene being tested, the amount of TYLCV DNA increased with F1 adult development, peaking at 11 DAE, and then decreased (Fig. 1*A*). Absolute quantification of viral DNA further confirmed the increase of TYLCV DNA in F1 adults during the first 11 DAE (*SI Appendix*, Fig. S1 *A* and *B*). The amount of viral CP in F1 adults of viruliferous whiteflies was monitored by Western blot using an anti-CP monoclonal antibody (25). Consistent with the dynamics of viral DNA, the level of TYLCV CP increased at 11 DAE and then decreased at 21 DAE (Fig. 1*B*). Previous studies have shown that cotton is a nonhost plant for TYLCV and that nonviruliferous whiteflies are unable to acquire TYLCV from cotton previously exposed to viruliferous whiteflies (26, 27). To further confirm the nonhost status of the cotton used in our study, young cotton plants were inoculated by exposing each plant to 20 viruliferous whiteflies for 48 h. qPCR analysis showed that no virus DNA was detected in newly emerged leaves of cotton plants at either 20 or 30 d postinoculation (dpi) (*SI Appendix*, Fig. S1*C*). Therefore, the adult offspring could not have acquired the virus from cotton plants, and the increase of TYLCV up to 11 DAE was most likely due to viral replication in the F1 adults.

To investigate the possible replication site(s) of TYLCV in whiteflies, we used fluorescence in situ hybridization (FISH) to visualize the VS DNA strand of TYLCV in MGs and PSGs, the two most important organs involved in virus transmission (28). FISH was performed by using F1 adults of viruliferous MEAM1 whiteflies with a fluorescent probe to the CP gene (*V1*). Surprisingly, whereas TYLCV VS DNA was found in the PSGs of F1 adults throughout their lives, no VS DNA was detected in the MGs of F1 adults (Fig. 1*C* and *SI Appendix*, Table S1). Similar results were obtained by immunostaining assays using the anti-CP monoclonal antibody (Fig. 1*D* and *SI Appendix*, Table S1). No specific signal of TYLCV VS DNA or CP was observed in MGs and PSGs from nonviruliferous MEAM1 whiteflies (*SI Appendix*, Fig. S1*E*).

### TYLCV Accumulates in the PSGs of MEAM1 Whiteflies during Long-Term Retention.

**Fig. 2.**
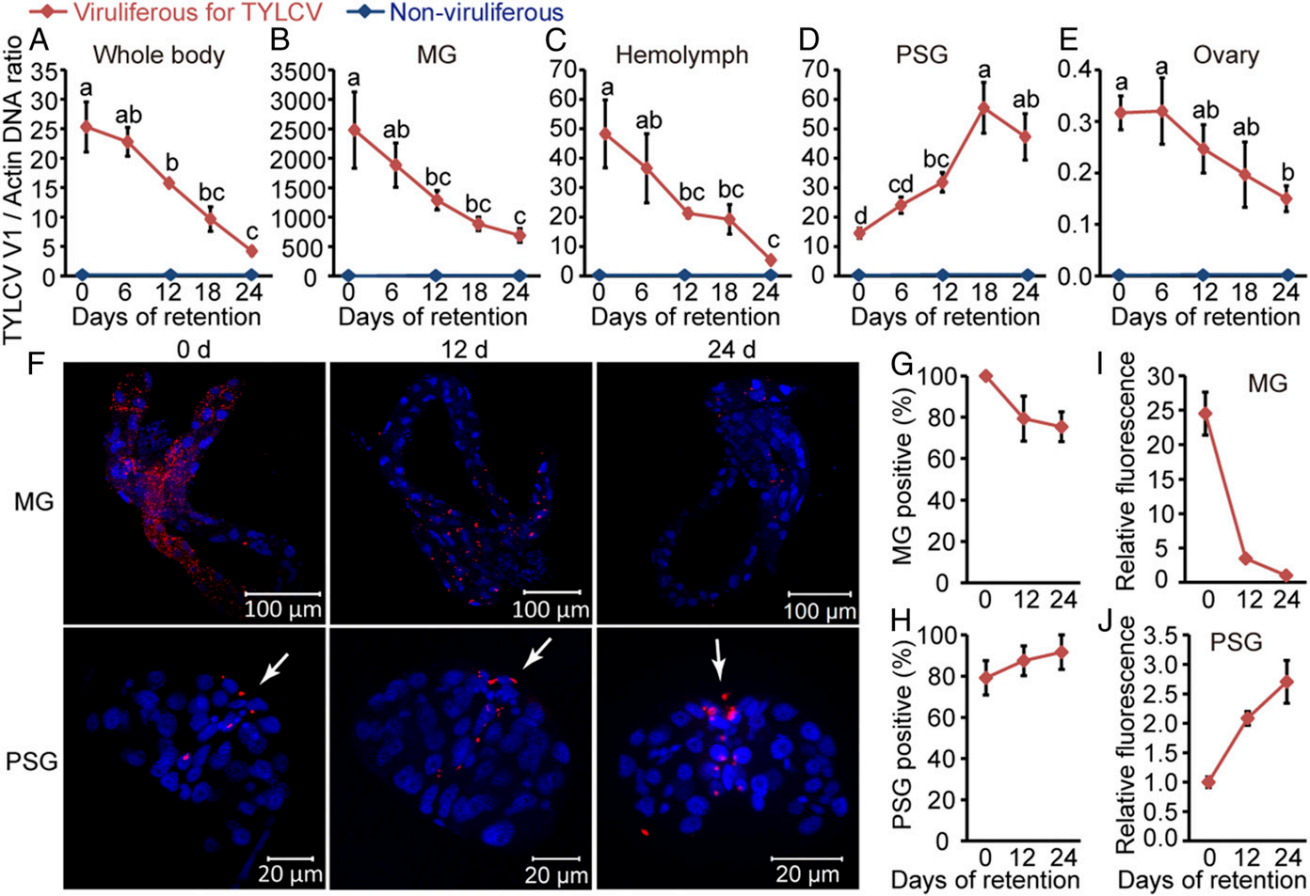
Dynamics of TYLCV in whole body and various tissues of MEAM1 whiteflies during long-term retention. (*A*–*E*) Relative concentration of TYLCV DNA in the whole body (*A*), MG (*B*), hemolymph (*C*), PSG (*D*), and ovary (*E*) of MEAM1 whiteflies. Total DNA was extracted from the whole body, MG, hemolymph, PSG, and ovary for assay by qPCR. Mean ± SEM of three independent experiments is shown. *P* < 0.05 (one-way ANOVA, least significant difference [LSD] test). (*F*) Localization of TYLCV VS DNA in MGs and PSGs of whiteflies after different times of retention. MGs and PSGs were hybridized with a Cy3-labeled VS strand-specific probe (V1 probe; red). Cell nucleus was stained with DAPI (blue). The white arrow indicates TYLCV VS DNA signal in the PSG. (*G* and *H*) The proportion of TYLCV-positive MGs (*G*) and PSGs (*H*) at each time point. MGs, *n* = 24; PSGs, *n* = 24. (*I* and *J*) Relative fluorescence density of TYLCV VS DNA signal in MGs (*I*) and PSGs (*J*). For MGs and PSGs, the fluorescence density was set at one at days 24 and 0, respectively. (*G*–*J*) Mean ± SEM of three independent experiments is shown.

The above results indicate that TYLCV may replicate mainly in the PSGs of whiteflies. However, another possibility is that the transovarially inherited virus might not enter into the MG, which could explain why it was not detected in this organ. To examine whether TYLCV also replicates in the MG and other tissues, we fed MEAM1 whiteflies on TYLCV-infected tomato plants for a 48-h acquisition-access period (AAP), a time sufficient for the virus to move through the whitefly body, from the MG into the hemolymph and, finally, into the PSG and ovary (14, 24). Then, the whiteflies were transferred onto cotton plants for long-term retention. Next, we used qPCR to examine the dynamics of viral DNA load in the whole body and various tissues after 0, 6, 12, 18, and 24 d of retention. The DNA load in the whitefly whole body decreased with the time (Fig. 2*A*). The viral load in MG, hemolymph, and ovary also decreased over time (Fig. 2 *B*, *C*, and *E*). In contrast, the viral load in the PSGs increased over time, peaking at 18 d after the whiteflies were transferred to cotton, and then decreased slightly (Fig. 2*D*). We further visualized the VS DNA strand in the MG and PSGs at 0, 12, and 24 d after transfer of whiteflies onto cotton. The proportion of TYLCV-positive MGs declined gradually (from 100 to 75%) after transfer to cotton, and the intensity of the viral signal decreased by 86% after 12 d of retention (Fig. 2 *F*, *G*, and *I*). However, the proportion of TYLCV-positive PSGs increased gradually (from 79 to 92%) over time, along with the intensity of the viral signal in PSGs (Fig. 2 *F*, *H*, and *J*). Similar results were obtained by immunostaining assays with the anti-CP monoclonal antibody (*SI Appendix*, Fig. S2 *A*–*C*). The specific accumulation of TYLCV in the PSGs is consistent with the hypothesis that TYLCV replicates mainly in the PSGs of whiteflies. However, translocation and accumulation of virus from other tissues into the PSGs during long-term retention may also lead to viral accumulation in the PSGs.

To examine whether virus translocation could lead to viral accumulation in the PSGs, we investigated the dynamics of another begomovirus, papaya leaf curl China virus (PaLCuCNV) (29), in the MEAM1 whitefly whole body and various tissues during long-term retention. Whiteflies were first fed on PaLCuCNV-infected tomato plants for a 48-h AAP and then transferred onto cotton, a nonhost plant for PaLCuCNV (*SI Appendix*, Fig. S1*D*). qPCR analysis showed that PaLCuCNV DNA loads in the whitefly whole body, MG, hemolymph, and PSG declined rapidly over time following transfer to cotton plants (*SI Appendix*, Fig. S2 *D*–*G*). Immunostaining assays with the same anti-CP monoclonal antibody showed that the proportion of PaLCuCNV-positive MGs decreased gradually (from 100 to 75%) over time (*SI Appendix*, Fig. S2 *H* and *I*). All PSGs were PaLCuCNV-positive immediately after the 48-h AAP, indicating that the virus is able to infect whitefly cells and translocate efficiently from the MG into the hemolymph and, finally, into the PSG. However, the proportion of PaLCuCNV-positive PSGs decreased from 100 to 9% after 12 d of retention and to none after 24 d (*SI Appendix*, Fig. S2 *H* and *J*). Taken together, these results indicate that virus translocation does not lead to its accumulation in the PSGs during long-term retention. Therefore, the accumulation of TYLCV in whitefly PSG was more likely due to replication rather than translocation.

### TYLCV Replicates in the PSGs of MEAM1 Whiteflies.

To verify the replication hypothesis, total DNA from whole whiteflies was used for Southern blot hybridization analysis. The dsDNA-replicative form of TYLCV was detected in viruliferous whiteflies, whereas only the ssDNA form of PaLCuCNV was detected in viruliferous whiteflies after retention (*SI Appendix*, Fig. S3 *A*–*D*). We then used two-step anchored qPCR (30) to examine the dynamics of the CS DNA strand of TYLCV, a marker of the dsDNA-replicative intermediates (9), in whole whiteflies and various tissues during long-term retention. The CS DNA was detected in the whole body, MG, and PSG of viruliferous whiteflies. Moreover, it decreased over time in the whole body and MG, whereas it increased gradually in the PSG, peaking at 18 d after transfer of the whiteflies to cotton, and then decreased (*SI Appendix*, Fig. S4 *A*–*E*). We also used FISH with a fluorescent probe to the Rep gene (*C1*) to visualize the CS DNA in MGs and PSGs of viruliferous whiteflies at the indicated time points during the long-term retention period. Fluorescent signal corresponding to CS DNA was only detected in the MGs of whiteflies immediately after the 48-h AAP, whereas some signal was found in the PSGs throughout the retention period (*SI Appendix*, Fig. S4*F* and Table S2). No CS DNA signal was detected in the MGs and PSGs from nonviruliferous whiteflies by FISH (*SI Appendix*, Fig. S4*G*). We then examined PaLCuCNV CS DNA in whole whiteflies and various tissues during long-term retention using the two-step anchored qPCR. PaLCuCNV CS DNA was only detected in the whole body and MGs of whiteflies immediately after the 48-h AAP, and no CS DNA was detected in the PSGs at any time point (*SI Appendix*, Fig. S5 *A*–*C*). FISH assays with a fluorescent probe to the *C1* gene did not reveal PaLCuCNV CS DNA in the PSGs of viruliferous whiteflies at any time point, and specific signal was only detected in the MGs of whiteflies immediately after the 48-h AAP (*SI Appendix*, Fig. S5*D* and Table S2).

**Fig. 3.**
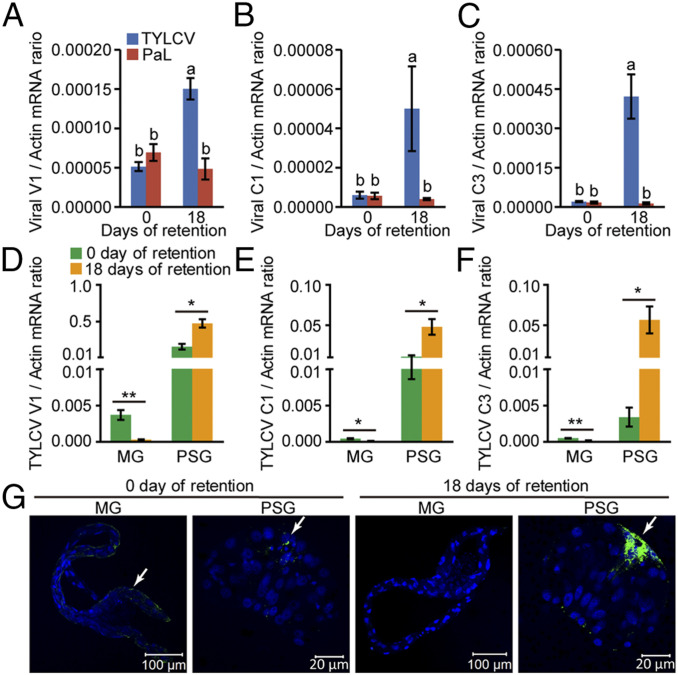
Expression of viral genes in MEAM1 whiteflies during long-term retention. (*A*–*C*) Relative expression levels of *V1* (*A*), *C1* (*B*), and *C3* (*C*) of TYLCV and PaLCuCNV (PaL) in whitefly whole bodies after 0 and 18 d of retention as detected by qRT-PCR. Mean ± SEM of three independent experiments is shown. *P* < 0.05 (one-way ANOVA, LSD test). (*D*–*F*) Relative expression levels of TYLCV *V1* (*D*), *C1* (*E*), and *C3* (*F*) genes in MGs and PSGs of whiteflies after 0 and 18 d of retention as detected by qRT-PCR. Mean ± SEM of three independent experiments is shown. *P < 0.05; **P < 0.01 (independent-sample *t* test). (*G*) Localization of TYLCV Rep in MGs and PSGs of whiteflies after 0 and 18 d of retention. Rep was detected by use of a rabbit anti-Rep polyclonal antibody and goat anti-rabbit IgG labeled with Dylight 488 (green) secondary antibody. Cell nucleus was stained with DAPI (blue). The white arrow indicates the immune-reactive signal of TYLCV Rep. For each time point, 20 samples were analyzed, and a similar trend was observed.

Next, the transcription of viral *V1*, *C1*, and *C3* genes in viruliferous whiteflies after 0 and 18 d of retention was monitored with qRT-PCR. TYLCV transcripts in whole whiteflies increased after 18 d of retention, whereas PaLCuCNV transcripts remained low (Fig. 3 *A*–*C*), indicative of active gene transcription of TYLCV, but not PaLCuCNV, during retention. We further examined TYLCV transcripts in the MGs and PSGs of whiteflies and found that the expression of all three genes was significantly higher in the PSGs than in the MGs. Moreover, TYLCV transcripts in the PSGs increased after 18 d of retention, but were reduced to undetectable levels in the MGs (Fig. 3 *D*–*F*).

To further confirm the expression of TYLCV proteins in the PSGs, mass spectrometry was performed to identify the viral peptides in total protein extracts of 1,000 PSGs of MEAM1 whiteflies after 18 d of retention on cotton. Many peptides corresponding to TYLCV-encoded proteins were identified in the PSGs from whiteflies viruliferous for TYLCV, whereas only the CP protein was detected in the PSGs from whiteflies viruliferous for PaLCuCNV (*SI Appendix*, Fig. S6 and Dataset S1). No viral peptides were detected in PSGs from nonviruliferous whiteflies. We also performed immunostaining assays in MGs and PSGs using an anti-TYLCV Rep antibody (31) to detect the Rep protein (*SI Appendix*, Fig. S7*A*), which is required for viral replication (9). The Rep signal was only detected in some MGs of whiteflies immediately after the 48-h AAP, whereas specific signal was found in the PSGs at both 0 and 18 d of retention, and the amount of Rep increased at 18 d of retention (Fig. 3*G* and *SI Appendix*, Table S3). No Rep signal was detected in the MGs and PSGs of nonviruliferous whiteflies (*SI Appendix*, Fig. S7*B*).

Southern blot hybridization analysis of total DNA from 11 DAE F1 adults derived from viruliferous MEAM1 whiteflies also validated the existence of the replicative dsDNA of TYLCV (*SI Appendix*, Fig. S3*E*). Localization of TYLCV CS DNA and Rep in the PSGs of F1 adults further supported replication of TYLCV in the PSG (*SI Appendix*, Fig. S7 *C* and *D*). Overall, these results demonstrated that TYLCV replicates mainly in the PSG of whiteflies, whereas PaLCuCNV is unable to replicate in whiteflies, even though it does infect whitefly cells.

### Transcriptional Response of MEAM1 Whitefly PSG to Virus Infection.

To gain further insight into the molecular mechanisms underlying the replication of TYLCV in whitefly PSGs, we used RNA-sequencing (RNA-seq) to investigate the transcriptional response of whitefly PSGs to virus infection. We found 2,070 up-regulated and 1,505 down-regulated genes in the PSGs of whiteflies viruliferous for TYLCV and 1,770 up-regulated and 1,920 down-regulated genes in the PSGs of whiteflies viruliferous for PaLCuCNV, compared to those of nonviruliferous whiteflies (Dataset S2). Functional analysis of differentially expressed genes showed that a number of genes involved in cell-cycle regulation were differentially expressed in the PSGs of viruliferous whiteflies, with more up-regulated in whiteflies viruliferous for TYLCV and more down-regulated in whiteflies viruliferous for PaLCuCNV. More genes involved in DNA replication and DNA repair were up-regulated in the PSGs of whiteflies viruliferous for TYLCV, but not in those viruliferous for PaLCuCNV (*SI Appendix*, Fig. S8*A* and Datasets S3 and S4). In plants, geminiviruses reprogram cell-cycle controls to induce the accumulation of host DNA synthesis machinery to support their replication (11, 32). The accumulation of viral DNA replication products and intermediates then triggers a genotoxic stress response and the synthesis of host DNA-repair proteins (33, 34). Thus, the RNA-seq data indicate that TYLCV may use similar mechanisms to replicate in both plant and insect hosts.

Several genes involved in the phosphoinositide 3-kinase–Akt, mitogen-activated protein kinase, transforming growth factor beta, and apoptosis signaling pathways were also differentially expressed in the PSGs of viruliferous whiteflies. Interestingly, more genes were induced by TYLCV, whereas more genes were suppressed by PaLCuCNV (*SI Appendix*, Fig. S8*B* and Datasets S3 and S4). In addition, many genes related to immune responses, such as lysosome, phagosome, and ubiquitin-mediated proteolysis, were up-regulated in the PSGs of viruliferous whiteflies (*SI Appendix*, Fig. S8*C* and Datasets S3 and S4), indicating that these immune systems are activated by virus infection and may contribute to virus degradation in the PSG. The RNA-seq data were confirmed by qRT-PCR analysis of 32 TYLCV-responsive genes and 22 PaLCuCNV-responsive genes related to the above processes (Dataset S5).

### TYLCV Induces and Recruits Whitefly PCNA for Its Replication.

**Fig. 4.**
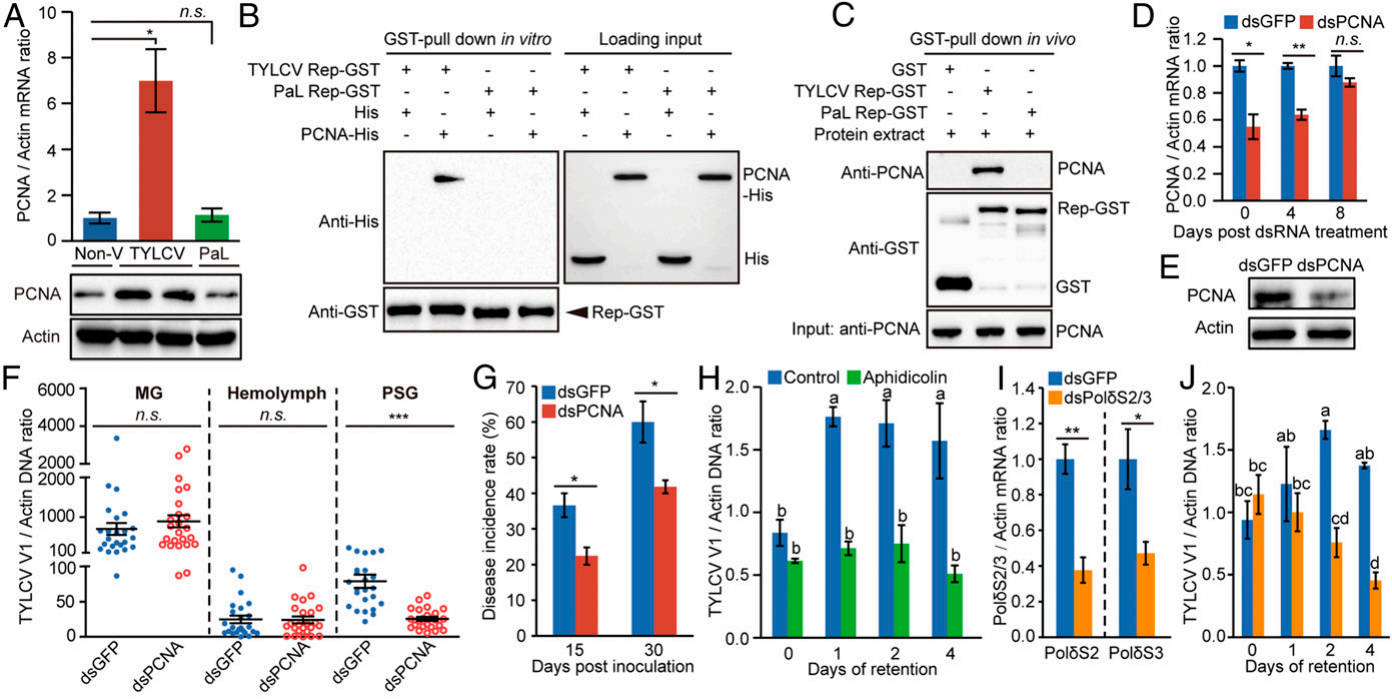
The function of whitefly PCNA and DNA Polδ in TYLCV replication. (*A*) PCNA expression was induced by TYLCV infection, but not by PaLCuCNV (PaL). Non-V, nonviruliferous. (*B* and *C*) Both recombinant PCNA (*B*) and whitefly endogenous PCNA (*C*) interacted with GST-fused TYLCV Rep, but not with GST-fused PaL Rep. (*D*) PCNA mRNA levels in whiteflies at several time points after dsRNA treatment. (*E*) PCNA protein levels in whiteflies at 0 d after dsRNA treatment. (*F*) dsPCNA treatment decreased TYLCV load in PSGs, whereas it did not affect the loads in the MGs and hemolymph. One dot represents one MG, PSG, or the hemolymph of one female whitefly. Mean ± SEM is shown. n.s., not significant. ***P < 0.001 (nonparametric Mann–Whitney *U* test). The results were reproduced at least two times. (*G*) The disease incidence rate of the tomato plants with TYLCV fed upon by dsPCNA-/dsGFP-treated whiteflies. (*H*) The effect of aphidicolin treatment on TYLCV replication in whiteflies. (*I*) Relative mRNA levels of DNA Polδ subunit 2 (PolδS2) and subunit 3 (PolδS3) after feeding with the mixture of dsPolδS2 and dsPolδS3. (*J*) The effect of dsPolδS2/3 treatment on TYLCV replication in whiteflies. (*A*, *D*, *G*, and *I*) Mean ± SEM of three independent experiments is shown. n.s., not significant. *P < 0.05; **P < 0.01 (independent-sample *t* test). (*H* and *J*) Mean ± SEM of three independent experiments is shown. *P* < 0.05 (one-way ANOVA, LSD test).

Proliferating cell nuclear antigen (PCNA) is a key host DNA synthesis protein that interacts with a variety of proteins involved in DNA replication, DNA repair, and cell-cycle regulation (35–37). Interestingly, the level of PCNA was up-regulated in the PSGs of whiteflies viruliferous for TYLCV, but not in those of whiteflies viruliferous for PaLCuCNV (Dataset S2). The trend was confirmed by qRT-PCR and on immunoblots of whitefly proteins (Fig. 4*A*). Given that Rep has been shown to interact with PCNA in plants (38, 39), we tested whether Rep also interacts with whitefly PCNA using pull-down assays. An in vitro glutathione *S*-transferase (GST) pull-down assay showed that GST-fused TYLCV Rep bound to His-tagged PCNA, whereas no binding was detected for a GST-fused PaLCuCNV Rep (Fig. 4*B*). An in vivo GST pull-down assay further showed that endogenous whitefly PCNA coeluted with GST-fused TYLCV Rep, but not with GST-fused PaLCuCNV Rep or GST alone (Fig. 4*C*). These findings suggest that the specific interaction between TYLCV Rep and whitefly PCNA is involved in virus replication in whiteflies.

To verify the role of PCNA in TYLCV replication in whiteflies, we knocked down PCNA expression using RNA interference (RNAi). Whiteflies were first fed on TYLCV-infected tomato plants for 48 h and then on dsRNA corresponding to PCNA or green fluorescent protein (GFP) (control) for another 48 h. After dsRNA treatment, whiteflies were transferred to cotton, and the transcript level of PCNA was monitored. Compared to whiteflies fed with double-stranded GFP (dsGFP), PCNA was silenced by 45% and 36% at days 0 and 4 after double-stranded PCNA (dsPCNA) treatment, respectively. At 8 d after dsRNA treatment, the level of PCNA was only slightly decreased in dsPCNA-treated whiteflies, indicating that the silencing effect nearly disappeared (Fig. 4 *D* and *E*). To show the cumulative effect of PCNA repression on TYLCV replication in whiteflies, the TYLCV load in whitefly MGs, hemolymph, and PSGs was quantified at 8 d after dsRNA treatment. Compared with the control group, dsPCNA-treated whiteflies showed similar virus load in the MG and hemolymph, but significantly lower virus load in the PSG (Fig. 4*F*). Virus-transmission assays showed that virus-transmission rates were lower for dsPCNA-treated whiteflies versus dsGFP-treated whiteflies (Fig. 4*G*). At 15 dpi, 36% of the plants in the control group showed TYLCV symptoms, whereas only 22% plants in the dsPCNA-treated group showed symptoms. At 30 dpi, 60% of the plants in the control group were symptomatic, whereas only 41% in the dsPCNA-treated group developed symptoms. In contrast, the transmission efficiency of PaLCuCNV was unaffected by dsPCNA treatment under the same conditions, though the overall transmission efficiency was considerably lower (*SI Appendix*, Fig. S9*A*). Taken together, these results indicate that the induction and recruitment of whitefly PCNA is important for virus replication in whiteflies.

### TYLCV Relies on Whitefly DNA Polymerase δ for Its Replication.

Like other small DNA viruses, geminiviruses do not encode their own DNA polymerases and, instead, depend on host polymerases for viral DNA synthesis. In animal and fungal cells, PCNA associates with DNA polymerase δ (Polδ) to promote processivity of the enzyme (40). Thus, we examined if whitefly DNA Polδ has a role in TYLCV replication using aphidicolin, which specifically inhibits the nuclear replicative DNA polymerases, Polα, δ, and ε, in eukaryotic cells (41). Previous studies have shown that the level of TYLCV in whole whiteflies increased during the first few days after a short-time AAP (20, 21). Given the limited efficacy period of aphidicolin, its effect on viral DNA accumulation in whole whiteflies was assessed after a 4-d retention period following a 6-h AAP (21). Whiteflies were first treated with aphidicolin and then allowed to feed on TYLCV-infected tomato plants for 6 h, followed by transfer onto cotton plants. At the time of transfer, the viral load in whole insects was similar between the treatment and control groups (Fig. 4*H* and *SI Appendix*, Fig. S9*B*), suggesting that aphidicolin did not affect whitefly feeding behavior. However, after 1 to 4 d of retention, the TYLCV burden in aphidicolin-treated whiteflies was significantly lower than in the control (Fig. 4*H*). In contrast, the burden of PaLCuCNV in whole insects decreased upon transfer onto cotton and was unaffected by aphidicolin treatment (*SI Appendix*, Fig. S9*B*).

To specifically assess the role of DNA Polδ in TYLCV replication, we knocked down the expression of whitefly DNA Polδ subunits 2 (PolδS2) and 3 (PolδS3) using RNAi. Compared with whiteflies fed with dsGFP, the messenger RNA (mRNA) levels of PolδS2 and PolδS3 were reduced by 62% and 53%, respectively, in whiteflies fed with the mixture of dsPolδS2 and dsPolδS3 for 48 h (Fig. 4*I*). After dsRNA treatment, whiteflies were allowed to feed on TYLCV-infected tomato plants for 6 h and then transferred onto cotton. At the time of transfer, the viral load in whole insects was similar between the dsPolδS2/3- and dsGFP-treated whiteflies (Fig. 4*J* and *SI Appendix*, Fig. S9*C*). However, the TYLCV load was significantly lower in the dsPolδS2/3-treated whole whiteflies compared with the dsGFP-treated whiteflies after 2 and 4 d of retention (Fig. 4*J*). As expected, the PaLCuCNV load in whole insects was unaffected by the dsPolδS2/3 treatment under the same conditions (*SI Appendix*, Fig. S9*C*). qRT-PCR analyses further showed that PolδS2 and PolδS3 transcripts were both significantly induced in whiteflies viruliferous for TYLCV, but not in whiteflies viruliferous for PaLCuCNV, when compared to nonviruliferous whiteflies (*SI Appendix*, Fig. S9 *D* and *E*), indicating that TYLCV can specifically induce the accumulation of whitefly DNA Polδ. Overall, these results demonstrate that TYLCV relies on DNA Polδ for its replication in whiteflies.

### Replication in PSG Contributes to Viral Infectivity Persistence after Long-Term Retention.

**Fig. 5.**
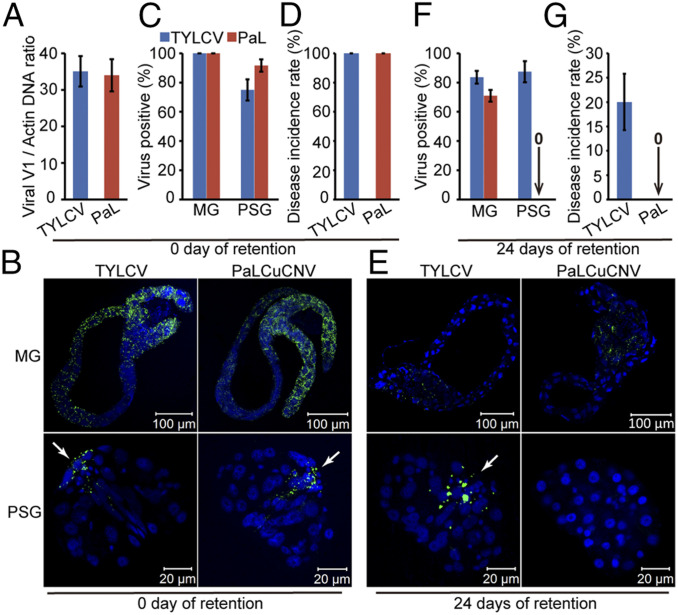
Replication of TYLCV in whitefly PSGs contributes to viral infectivity persistence after long-term retention. (*A*) Relative abundance of TYLCV and PaLCuCNV (PaL) in whitefly whole bodies immediately after a 48-h AAP on TYLCV- or PaL-infected tomato plants. (*B*) Localization of TYLCV or PaL in MGs and PSGs of whiteflies immediately after the 48-h AAP. (*C*) The proportion of TYLCV- or PaL-positive MGs and PSGs immediately after the 48-h AAP. MGs, *n* = 24; PSG, *n* = 24. (*D*) The disease incidence rate of the tomato plants with TYLCV or PaL fed upon by whiteflies that immediately after the 48-h AAP. (*E*) Localization of TYLCV or PaLCuCNV in MGs and PSGs of whiteflies after 24 d of retention. (*F*) The proportion of TYLCV- or PaL-positive MGs and PSGs after 24 d of retention. MGs, *n* = 24; PSGs, *n* = 24. (*G*) The disease incidence rate of the tomato plants with TYLCV or PaL fed upon by whiteflies after 24 d of retention. (*B* and *E*) TYLCV and PaLCuCNV CP was detected by use of a mouse anti-CP monoclonal antibody and goat anti-mouse IgG labeled with Dylight 488 (green) secondary antibody. Cell nucleus was stained with DAPI (blue). The white arrow indicates the viral CP signal in the PSG. For each time point, 24 samples were analyzed, and a similar trend was observed. (*A*, *C*, *D*, *F*, and *G*) Mean ± SEM of three independent experiments is shown.

To examine the effect of viral replication in whitefly PSGs on virus spread, we compared the transmission efficiency of TYLCV and PaLCuCNV by MEAM1 whiteflies. Whiteflies were allowed to feed on TYLCV- or PaLCuCNV-infected tomato plants for 48 h. Half of the insects were collected immediately, and the other half were transferred onto cotton plants and collected after 24 d of retention. The amount of TYLCV ingested by whiteflies was comparable to that of PaLCuCNV after the 48-h AAP (Fig. 5*A*). Immunostaining assays using the anti-CP antibody showed that all MGs were positive for both viruses, and 75% and 92% of PSGs were positive for TYLCV or PaLCuCNV, respectively (Fig. 5 *B* and *C*). Virus-transmission assays showed that all 30 plants whitefly-inoculated with TYLCV or PaLCuCNV became infected after a 48-h inoculation access period (IAP) (Fig. 5*D*). After 24 d of retention, 83% of MGs and 88% of PSGs were positive for TYLCV (Fig. 5 *E* and *F*). In contrast, 70% of MGs were positive for PaLCuCNV, but none of the PSGs contained detectable virus. In subsequent virus-transmission assays, 6 of 30 plants were infected in the TYLCV treatment, whereas none of the 30 plants was infected in the PaLCuCNV treatment (Fig. 5*G*), suggesting that replication of TYLCV in the PSG contributes to viral infectivity persistence after long-term retention.

### Replication of TYLCV in PSGs of MED Whiteflies.

Finally, we examined whether TYLCV also replicates in the PSGs of MED whiteflies, another invasive cryptic species of the *B. tabaci* complex (12). MED whiteflies were fed on TYLCV-infected tomato plants for 48 h and then transferred onto cotton. The proportion of TYLCV-positive MGs decreased gradually (from 75 to 54%) after transfer. However, the proportion of TYLCV-positive PSGs increased gradually (from 58 to 83%) over time (*SI Appendix*, Fig. S10 *A*–*C*). Moreover, TYLCV CS DNA and Rep were detected in the PSGs after 24 d of retention (*SI Appendix*, Fig. S10 *D* and *E*), suggesting that TYLCV also replicates in the PSGs of MED whiteflies.

## Discussion

A longstanding and important issue in the study of begomoviruses is whether these viruses replicate within the whitefly vector. This question is of major importance because replication of viruses in their vectors could impact virus spread and the physiology of the vectors (42). Lifetime infectivity persistence, alterations to whitefly biology, and accumulation of viral DNA and transcripts in the vector have been proposed as evidence of replication of TYLCV in the whitefly vector (17–21). However, contrasting evidence has been reported by two studies, suggesting that TYLCV does not replicate within whiteflies (22, 23). In these previous studies, time-course quantification of viral load using qPCR showed that TYLCV DNA loads within whole whiteflies remained stable or slightly decreased after viral acquisition had stopped. Thus, based on not detecting an increase of the VS and CS DNA strands in whole insects after 24- and 76-h AAP, Becker et al. (22) and Sánchez-Campos et al. (23) concluded that TYLCV did not replicate in its whitefly vector or that replication was below the limits of detection in their experiments. Previously, we demonstrated that TYLCV is transmitted transovarially in MEAM1 whiteflies and efficiently (68 to 92%) reaches all developmental stages of the progeny and that the adult offspring can transmit the virus to tomato plants (24). Here, we further determined that the amount of TYLCV delivered transovarially to F1 adults, which developed from eggs deposited on cotton by viruliferous MEAM1 whiteflies, increased during the first 11 DAE (Fig. 1 *A* and *B*). Consistent with cotton being a nonhost of TYLCV (26, 27), exposure of cotton plants to large numbers of viruliferous whiteflies did not result in symptomless TYLCV infection, based on failure to detect viral DNA in newly emerged leaves by the highly sensitive qPCR method (*SI Appendix*, Fig. S1*C*). Therefore, the cotton plant did not provide viral DNA to the adult offspring, and the increase of TYLCV up to 11 DAE was most likely due to viral replication in the F1 adults. The viral load in F1 adults decreased after 11 d of age, likely reflecting an effect of whitefly age on viral accumulation. The average lifespan of MEAM1 adults on cotton plants is about 30 d (43). A previous study reported that, after 10 d of age, the amount of TYLCV that accumulated in whiteflies following a 48-h AAP rapidly decreased, such that 17-d-old whiteflies contained less than half as much virus compared to 10-d-old whiteflies. At the age of 24 d, this amount was reduced to 10% and was barely detectable thereafter (17). Thus, the change in whitefly physiological status after 11 d of age may lead to the decrease of TYLCV load in F1 adults of viruliferous whiteflies.

Several lines of evidence suggest that TYLCV replicates mainly in whitefly PSGs. First, TYLCV DNA and CP were only detected in the PSGs of adult offspring derived from viruliferous whiteflies (Fig. 1 *C* and *D*). Second, TYLCV total DNA and the replicative CS DNA specifically accumulated in the PSGs during long-term retention following a 48-h AAP, whereas no such accumulation was found for PaLCuCNV under the same conditions (Fig. 2 and *SI Appendix*, Figs. S2–S5). Third, TYLCV transcripts in the PSGs increased after 18 d of retention, but were reduced to undetectable levels in the MGs (Fig. 3 *D*–*G*). This finding may help to explain why replication was not detected in whiteflies post-AAP when whole insects were analyzed (22, 23) (Fig. 2*A*). Our data indicate that the viral load in the MGs was far greater than that in other tissues after a 48-h AAP, especially the TYLCV DNA content in the PSGs, which was only about 1 to 5% of that in the MGs (Fig. 2 *A*–*E*). Hence, the limited replication of TYLCV in the PSGs was masked by the large amount of virus degraded in the MGs when whole insects were analyzed.

Our study showed that the TYLCV burden in dimethyl sulfoxide- or dsGFP-treated whole whiteflies increased during the first few days after a 6-h AAP (Fig. 4 *H* and *J*). Several groups have also observed a transient increase in TYLCV load in whole insects after a short AAP (17, 20, 21). Pakkianathan et al. (20) found that, following an 8-h AAP on TYLCV-infected plants or purified virions and transfer to cotton plants, the amount of TYLCV in whole whiteflies increased during the first few days and then decreased. However, imposing additional stress by exposing viruliferous whiteflies to insecticides or heat stress resulted in continuous virus accumulation or reduction in whiteflies, respectively. This report suggested that the whitefly immune system or another defense pathway suppresses the ability of the virus to replicate in the whitefly vector. This hypothesis was confirmed by Wang et al. (21), who showed that after a 6-h AAP on TYLCV-infected plants and transfer to cotton, the accumulation of TYLCV activates whitefly autophagy at 48 h after transfer, and activated autophagy leads to subsequent degradation of TYLCV. Moreover, they also showed that the whitefly autophagy is activated after a longer (24- to 72-h) AAP on TYLCV-infected plants (21). Here, we showed that TYLCV DNA load in the whitefly whole body decreased with the time after a 48-h AAP (Fig. 2*A*). Therefore, experimental timing can lead to different outcomes that may also help to explain the previous contradictory results (17–22). After a short AAP, the whitefly immune response is not activated, and viral replication results in an increase of TYLCV in whole insects. In contrast, when the whitefly immune system is already activated after a longer AAP, no initial accumulation of TYLCV would be detected in whole whiteflies.

Whereas TYLCV CS DNA was consistently detected in the whole body and PSGs of viruliferous whiteflies, PaLCuCNV CS DNA was only detected in the whole body and MGs immediately after a 48-h AAP (*SI Appendix*, Figs. S4 and S5), indicating that the initial detection of PaLCuCNV CS DNA was not associated with viral replication. Similar uptake of viral CS DNA with the phloem sap ingested by whiteflies feeding on virus-infected plants has been shown by primer extension followed by Southern blotting or by qPCR (17, 22). A plausible explanation for the presence of viral CS DNA in whiteflies immediately after viral acquisition is that a dsDNA form of the virus is ingested from the phloem, as opposed to only virions with VS DNA. However, it is important to point out that the presence of CS DNA in phloem sap has not yet been demonstrated.

It is important to note that our data do not exclude the possibility that TYLCV also replicates in tissues other than the PSG. The presence of TYLCV CS DNA and Rep in the MGs of whiteflies immediately after a 48-h AAP suggests that TYLCV may also replicate in the MG epithelial cells (Fig. 3*G* and *SI Appendix*, Fig. S4 *B* and *F*). These observations are consistent with the results of a previous study, which showed replication of TYLCV in the MGs of whiteflies after an 8-h AAP (20). The transovarial transmission of TYLCV implies that TYLCV may also replicate in the ovary. Our qPCR analyses showed that the signals from CS DNA in the ovaries of viruliferous whiteflies were significantly higher than in nonviruliferous whiteflies at 0 and 24 d of retention (*SI Appendix*, Fig. S4*E*), indicative of the presence of TYLCV CS DNA in the ovaries of viruliferous whiteflies. However, more detailed investigations are needed to conclude whether TYLCV replicates in these tissues.

Due to their restricted genome size, small DNA viruses do not encode the DNA polymerases and accessory factors required for their replication, and instead recruit the replication machinery from their host to establish a productive infection. In eukaryotic cells, the expression of DNA synthesis machinery is tightly regulated by cell-cycle and developmental controls (11). Mammalian DNA tumor viruses—e.g., simian virus 40, papillomavirus type 16, and adenovirus type 6—encode multifunctional regulatory proteins that cause the host cell to enter S phase and produce host DNA synthesis machinery necessary for virus replication (44, 45). In the present study, we found that a number of genes involved in cell-cycle regulation were differentially expressed in the PSGs of whiteflies viruliferous for TYLCV. Moreover, many genes involved in DNA replication were up-regulated in the PSGs of viruliferous whiteflies (*SI Appendix*, Fig. S8*A*). These data suggest that TYLCV may use the same strategy as other small DNA viruses to create a replication-competent environment in whiteflies. In plants, the geminivirus Rep protein binds to the host retinoblastoma-related protein (pRBR), a key regulator of the plant cell cycle, to disrupt the interaction between pRBR and E2F transcription factors, which induces the production of plant DNA synthesis machinery (46, 47). Whether TYLCV uses the same mechanism as in plants or has evolved novel strategies to reprogram whitefly processes needs further investigation.

PCNA, originally characterized as a DNA Polδ accessory protein, functions as a DNA sliding clamp for Polδ and is an essential component for eukaryotic chromosomal DNA replication (48). Subsequent studies revealed that PCNA interacts with multiple partners involved in DNA replication, DNA repair, and cell-cycle regulation, functioning as a docking partner that coordinates various protein interactions with DNA (48, 49). Here, we discovered that the interaction between TYLCV Rep and whitefly PCNA was important for virus replication in whiteflies (Fig. 4). TYLCV may use the interaction between Rep and PCNA to recruit DNA synthesis machinery in the whitefly vector, such as DNA Polδ, for its replication. In contrast, PaLCuCNV Rep did not interact with whitefly PCNA, further supporting the idea that the interaction between TYLCV Rep and whitefly PCNA is important for virus replication in whiteflies. Previous studies showed that geminivirus Rep bound to plant PCNA, and the interaction is thought to induce the assembly of the plant-replication complex close to the viral origin of replication (38, 39). Therefore, it seems that TYLCV Rep has evolved to interact with both the plant- and insect-encoded PCNA. Comparison of PCNA amino acid sequences of *B. tabaci* with *Nicotiana tabacum* and *Solanum lycopersicum*, hosts of TYLCV and PaLCuCNV, showed that *N. tabacum* PCNA shared 97.7% identity with *S. lycopersicum* PCNA, whereas *B. tabaci* PCNA shared only 66.3% and 65.5% identity with *N. tabacum* and *S. lycopersicum* PCNA, respectively (*SI Appendix*, Fig. S11*A*). Furthermore, there are 76 amino acid differences between the Reps of TYLCV and PaLCuCNV (*SI Appendix*, Fig. S11*B*). Future study of the critical amino acids in TYLCV Rep that are responsible for interacting with whitefly PCNA would shed light on the evolution of TYLCV to replicate in both plant and insect hosts.

The PSG is the final destination for virus circulating within the whitefly body before transmission to plants (14, 50). Replication of TYLCV in the PSG makes this organ a reservoir of virus and, thus, may contribute to virus spread. Our transmission assays showed that all test plants inoculated by whiteflies immediately after virus acquisition were infected by TYLCV or PaLCuCNV (Fig. 5*D*), indicating that replication is not required for efficient transmission. However, TYLCV infectivity was maintained in whiteflies after 24 d of retention, whereas PaLCuCNV was lost over the same time frame (Fig. 5*G*), suggesting that replication of TYLCV may contribute to viral persistence after long-term retention. Our findings provide insight into TYLCV’s lifetime infectivity persistence, which may help explain the global spread of TYLCV.

## Materials and Methods

### Vertical Transmission of TYLCV by MEAM1 Whitefly.

About 400 mixed-sex whiteflies (F0) were collected 8 to 10 DAE, moved to TYLCV-infected tomato plants for a 48-h AAP, and then transferred to cotton plants for 72 h (24). Then, adults were removed, and eggs were left on cotton leaves to develop. Following eclosion of the F1 adults, whiteflies were collected at 1, 6, 11, 16, 21, and 31 DAE for TYLCV DNA-load quantification. For each time point, 10 female whiteflies were used as one sample, and three replicates were examined. One hundred female adults at 1, 11, and 21 DAE were used for immunoblot analyses of TYLCV CP using an anti-CP monoclonal antibody (25). For TYLCV VS DNA and CP localization in F1 adults, MGs and PSGs were dissected from female whiteflies at 1, 11, and 31 DAE and used for FISH and immunofluorescence analysis. For each time point, 20 MGs and 20 PSGs were analyzed.

### Quantification of Virus DNA Load in Whitefly Whole Body and Various Tissues.

Newly emerged whiteflies were given a 48-h AAP on virus-infected or uninfected plants and then transferred to cotton, a nonhost plant of TYLCV and PaLCuCNV. The insects were transferred to new cotton plants after 2 wk to avoid emergence of new adults. Whiteflies were collected at 0, 6, 12, 18, and 24 d after the transfer. MGs, PSGs, and ovaries were dissected from single female whiteflies in prechilled phosphate-buffered saline (PBS) buffer and flushed several times with PBS to remove contaminating viruses. For hemolymph isolation, each whitefly was dissected from the abdomen in 5 μL of prechilled PBS buffer. Then, all of the liquid was collected without contamination from tissues. Because the insect fat body is suspended in the hemolymph, we collected the hemolymph and fat body together. For both viral total DNA- and CS DNA-load quantification at each time point, 10 whole whiteflies, 10 MGs, 10 PSGs, 10 ovaries, or the hemolymphs of 10 female whiteflies were collected as one sample, and three replicates were examined. Total DNA of whitefly whole bodies or tissues was extracted by using described methods (51) and used for virus-load quantification according to described methods in *SI Appendix, Supplementary Text*.

### FISH.

Whiteflies were sampled at the indicated time points. MGs and PSGs were dissected from female whiteflies and then fixed in Carnoy’s fixative (chloroform–ethanol–acetic acid [6:3:1]) for 5 min and hybridized overnight in hybridization buffer (20 mM Tris⋅HCl [pH 8.0], 0.9 M NaCl, 0.01% sodium dodecyl sulfate, and 30% formamide) containing 10 pmol of fluorescent probe per mL. The sequences of the probes used to localize the VS and CS DNA strands are detailed in Dataset S6. After extensive washing in PBS, the tissues were mounted in fluoroshield mounting medium with DAPI (Abcam, catalog no. ab104139) and imaged on a Zeiss LSM710 confocal microscope. Nonviruliferous whiteflies were used as controls.

### Immunostaining Assay.

Whiteflies were sampled at the indicated time points. MGs and PSGs were dissected from female whiteflies. The specimens were fixed in 4% paraformaldehyde (MultiSciences Biotech, catalog no. LK-F0001) for 1 h at room temperature and washed in Tris-buffered saline (TBS) containing 0.1% Triton X-100 three times. Then the specimens were blocked in TBST (TBS buffer with 0.05% Tween 20) containing 1% bovine serum albumin (BSA) (MultiSciences Biotech, catalog no. A3828) for 2 h at room temperature, followed by incubation with anti-CP monoclonal antibody (1:500) or anti-Rep (31) (Agrisera, catalog no. AS153055) polyclonal antibody (1:200) in TBST containing 1% BSA overnight at 4 °C and then with goat anti-mouse (1:500) or goat anti-rabbit (1:500) secondary antibody labeled with Dylight 488 (MultiSciences Biotech, catalog no. LK-GAM4882) or Dylight 549 (MultiSciences Biotech, catalog no. LK-GAR5492) in TBST containing 1% BSA for 1 h at room temperature after extensive washing. After extensive washing in TBST, the tissues were mounted in fluoroshield mounting medium with DAPI (Abcam, catalog no. ab104139) and imaged on a Zeiss LSM710 confocal microscope (Zeiss). Nonviruliferous whiteflies were used as controls.

### Gene Silencing by Oral Ingestion of dsRNA.

RNA silencing was performed as described (24). Briefly, dsRNAs were diluted into 15% (wt/vol) sucrose solution at the concentration of 200 ng/μL, and ∼100 whiteflies were released into each feeding chamber. The chamber was incubated in an insect-rearing room for 48 h. For PCNA silencing, newly emerged whiteflies were first given a 48-h AAP on virus-infected plants and then collected for dsRNA feeding. Subsequently, whiteflies were collected at 0, 4, and 8 d after dsRNA treatment, and total RNA was extracted from groups of 20 female individuals to examine the PCNA mRNA levels. Total protein was extracted from 50 female whiteflies at 0 d after dsRNA treatment to examine the PCNA protein level. The remaining insects were collected at 8 d after dsRNA treatment and used for quantitative assays and virus-transmission tests. For PolδS2 and PolδS3 silencing, a mixture of dsPolδS2 and dsPolδS3 was used to feed newly emerged whiteflies. Then, total RNA was extracted from groups of 20 female individuals to examine the mRNA levels of PolδS2 and PolδS3, and three replicates were conducted. The remaining insects were given a 6-h AAP on virus-infected plants and then transferred to cotton. Whiteflies were collected at 0, 1, 2, and 4 d after the transfer and used for viral-load quantification. For each time point, 10 female whiteflies were used as one sample, and three replicates were examined.

### Transmission of TYLCV and PaLCuCNV to Plants by Whiteflies.

For virus transmission to cotton plants, whiteflies were collected in groups of 20 (female:male = 1:1) after a 48-h AAP from virus-infected or uninfected tomato plants, and each group was used to inoculate one young cotton plant. The inoculation was performed on the top third leaf of the plant at the three- to four-true-leaf stage for a 48-h IAP, by using a leaf clip cage (52). Virus transmission to tomato plants using the same batch of whiteflies was used as a control. Whiteflies were collected in groups of 10 (female:male = 1:1) and used to inoculate one uninfected tomato plant as above. Five cotton plants and three tomato plants were used for each transmission test. For virus transmission by PCNA-knocked-down whiteflies, viruliferous insects were collected in groups of 10 (female:male = 1:1) at 8 d after dsRNA treatment, and each group was used to inoculate one uninfected tomato plant as above. For transmission comparison between TYLCV and PaLCuCNV, newly emerged whiteflies were caged with virus-infected tomato plants for a 48-h AAP. Half of the insects were collected in groups of 10 (female:male = 1:1) immediately after the 48-h AAP and used for inoculation as above. The other half was moved to cotton. The insects were transferred to new cotton plants after 2 wk to avoid emergence of new adults. After 24 d of retention, groups of 10 (female:male = 1:1) whiteflies were collected and used for inoculation. The plants were then sprayed with imidacloprid at a concentration of 20 mg/L to kill all of the whitefly adults and eggs and maintained in insect-proof cages at 26 °C (±1 °C) under a photoperiod of 14:10 h (light:dark) to observe disease symptoms. Ten plants per replicate and three replicates were used to calculate the disease incidence rate for each transmission test.

### Data Availability.

We have deposited the short-read sequence data of RNA-seq in the National Center for Biotechnology Information Sequence Read Archive (accession no. PRJNA597917).

## Supplementary Material

Supplementary File

Supplementary File

Supplementary File

Supplementary File

Supplementary File

Supplementary File

Supplementary File
