# Supplementary material for: A plant DNA virus replicates in the salivary glands of its insect vector via recruitment of host DNA synthesis machinery
Source: Proc Natl Acad Sci U S A. 2020 Jul 7;117(29):16928–37. doi: 10.1073/pnas.1820132117 (PMC7382290; doi:10.1073/pnas.1820132117)
Supplement: Supplementary File [file pnas.1820132117.sapp.pdf]

PNAS
www.pnas.org

Supplementary Information for

A plant DNA virus replicates in the salivary glands of its insect vector via recruitment of host DNA synthesis machinery

Ya-Zhou He, Yu-Meng Wang, Tian-Yan Yin, Elvira Fiallo-Olivé, Yin-Quan Liu, Linda Hanley-Bowdoin, Xiao-Wei Wang

Xiao-Wei Wang
Email: xwwang@zju.edu.cn

This PDF file includes:

- Supplementary text
- Figures S1 to S11
- Tables S1 to S3
- Legends for Datasets S1 to S6
- SI References

Other supplementary materials for this manuscript include the following:

- Datasets S1 to S6

www.pnas.org/cgi/doi/10.1073/pnas.1820132117

Supplementary Information Text

Insects, viruses and plants

Two cryptic species of the *Bemisia tabaci* whitefly complex, Middle East Asia Minor 1 (MEAM1) (mitochondrial cytochrome oxidase I; GenBank accession no. GQ332577) and Mediterranean (MED) (mitochondrial cytochrome oxidase I; GenBank accession no. GQ371165), were reared on cotton plants (*Gossypium hirsutum* L. cv. Zhemian 1793) in insect-proof cages at 26 ± 1 °C under a 14:10-h (light/dark) photoperiod and relative humidity of 50 ± 10%. The purity of the culture was monitored every three generations by amplifying and sequencing the mitochondrial cytochrome oxidase I gene, which has been used widely to differentiate *B. tabaci* genetic groups (1). Clones of TYLCV isolate SH2 (AM282874) and PaLCuCNV isolate HeNZM1 (FN256260) were agroinoculated onto tomato (*Solanum lycopersicom*. cv. Hezuo903). Plants were grown in insect-proof greenhouses under controlled temperature at 25 ± 3 °C and natural lighting supplemented with artificial lights for 14 h/day.

Quantification of virus DNA load in whitefly whole body and various tissues

We used a two-step quantitative PCR (qPCR) with anchored primers to specifically quantify the viral CS DNA strand (2). In the first step, reactions were performed in 10 μL containing 1 μM primer, 1% BSA and 1 μM dNTPs in T4 DNA polymerase buffer. Reaction mixes were denatured at 95 °C for 10 min, cooled to room temperature, and incubated at 37 °C for 30 min with 1 unit of T4 DNA polymerase (TaKaRa, 2040A). Following the reaction, primers were removed using the AxyPrep PCR Cleanup kit (Axygen, 19817KB1). In the second step, 2 μL of the first-strand reaction were used for qPCR. qPCR was performed using an ABI Prism 7500 Fast Real-Time PCR system (Applied Biosystems) with SYBR Premix Ex TaqTM II (TaKaRa, RR820A), and the primers are shown in Dataset S6. For each reaction, 0.8 μL of each primer (10 mM), 6.4 μL of nuclease-free water, 10 μL of SYBR Premix Ex Taq and 2 μL of DNA extract were added. The qPCR protocol was 95°C for 30 s, followed by 40 cycles of at 95°C for 5 s and 60°C for 30 s. The *β-actin* (GenBank accession no. AF071908) gene of *B. tabaci* was measured in parallel as an internal control. The relative abundance of viral total DNA or CS DNA was calculated using the $2^{-\Delta Ct}$ method. To eliminate background signal that comes from possible nonspecific amplification or primer dimer formation during qPCR, total DNA extracted from whole bodies and various tissues of non-viruliferous whiteflies was used as negative controls in qPCR. If a significant difference appears between the signal for viral DNA in tissues from viruliferous versus non-viruliferous whiteflies, it would be considered as virus DNA positive. Otherwise, the signal would be considered as background signal due to nonspecific amplification of whitefly genome DNA or formation of primer dimers.

For absolute quantification of TYLCV total DNA in adult offspring of viruliferous whiteflies, dilution series containing between 5×10^2 and 5×10^7 copies of circular dsDNA pGEM-T Easy Vector carrying a full-length copy of the viral genome were used as quantification standards. The number of TYLCV DNA molecules was calculated according to the generated standard curve.

Detection of viral DNA in plants and insects by Southern blot hybridization

For hybridization of virus DNA in plants, newly emerged leaves of tomato plants at three weeks after agroinoculation were collected for DNA extraction. Total DNA from newly emerged leaves of uninfected tomato plants was used as a control. For hybridization of virus DNA in insects, about 4000 mixed-sex whiteflies were collected 1-3 days after eclosion (DAE), moved to virus-infected or uninfected tomato plants for a 48-h AAP, and then transferred to cotton plants for one week before DNA extraction. About 3000 mixed-sex first generation (F1) adults, which developed from eggs deposited on cotton by TYLCV-infected or non-viruliferous whiteflies, that at 11 DAE were used for DNA extraction. Total DNA from tomato plants and whole insects was extracted using previously described methods (3). Genomic DNA was stained using ethidium bromide as a loading control. After depurination and neutralization, total DNA was transferred to Hybond N^+ nylon membranes (Amersham) by upward capillary transfer in 0.4 M NaOH solution. The membranes were hybridized at 65°C with [α-^{32}P]dCTP labeled DNA probe corresponding to virus genomic DNA. A 910-bp fragment of TYLCV genomic DNA or a 991-bp fragment of PaLCuCNV genomic DNA was used as template for synthesis of [α-^{32}P]dCTP labeled DNA probe with the Random primer DNA labeling kit (TaKaRa, 6045) following manufacturer's instructions, respectively.

RNA isolation and quantitative reverse transcription PCR (qRT-PCR) analysis

For gene expression level measurement, total RNA was isolated from groups of twenty female whiteflies, fifty MG or fifty PSG using TRIzol reagent (Ambion, 15596018), and cDNAs were produced using the PrimeScript RT reagent kit with gDNA Eraser (TaKaRa, RR047A) following manufacturer's instructions. To assess viral gene transcripts in MG and PSG, TYLCV *V1, C1, C3* and *B. tabaci β-actin* were further amplified using Single Cell Sequence Specific Amplification Kit (Vazyme, P621-01) with gene specific primers (*SI Appendix*, Dataset S6) following manufacturer's instructions, omitting the reverse transcription step. For each reaction, 5 μL of reaction buffer, 1 μL of primer pool (0.1 μM), and 0.2 μL of RT/Taq enzyme were added, in a total volume of 10 μL. Reaction mixes were subjected to one cycle at 95 °C for 3 min, twelve cycles at 95 °C for 15 s and 60 °C for 15 min. The products were then used for qPCR with the appropriate primer pair combination after proper dilution. The qPCR results were normalized to the *B. tabaci β-actin* gene. The relative gene expression level was calculated using the $2^{-\Delta Ct}$ method.

Identification of virus-encoded proteins in PSG by mass spectroscopy

About 1000 PSG were dissected from whiteflies after 18 days of retention following a 48-h AAP on virus-infected or uninfected tomato plants, respectively. Total protein was extracted using the Minute total protein extraction kit (Invent, SD-001), and the concentration of the dissolved peptide solution was analyzed by A280 absorption using a NanoDrop UV–vis spectrophotometer (Thermo Fisher Scientific). Then, 50 μg proteins were digested with trypsin (Sigma) according to a filter aided sample preparation method (4), and the peptides were subjected to liquid chromatography-tandem MS spectroscopy analysis. Peptides were identified using an LTQ-Orbitrap Velos Pro with Easy nanoLC 1000 (Thermo Fisher Scientific), and proteomics data were analyzed using Proteome Discoverer 1.4 workflow (Thermo Fischer Scientific, Bremen, Germany).

RNA sequencing analysis

Given the small size of whitefly PSG, we used the single cell RNA sequencing method to assess transcriptional response of whitefly PSG to virus infection. Newly emerged whiteflies were first given a 48-h AAP on TYLCV-infected, PaLCuCNV-infected or uninfected tomato plants. Then, PSG were dissected from female whiteflies. Complementary DNA (cDNA) was prepared from PSG, amplified with SMART-Seq2 protocol (5), and subjected to library construction for transcriptome analysis using the Illumina HiSeq platform at Annoroad Gene Technology Company (Beijing, China). After removing adaptor sequences, empty reads and low quality sequences, the clean reads were aligned to the *B. tabaci* MEAM1 genome database (http://www.whiteflygenomics.org/) using TopHat (v2.0.12) (6). For gene expression analysis, reads per kilobase million mapped reads (RPKM) were calculated to estimate the expression level of genes in each sample (7). DEGseq (v1.18.0) was used to identify differentially expressed gene (DEG) between viruliferous PSG and non-viruliferous PSG. Genes with $q \leq 0.05$ (adjusted p-value) and $\log_2$ ratio ≥ 1 were considered differentially expressed. Web GO functional annotation plot and KEGG pathway database were used to investigate the functions of DEGs.

GST pull-down and western blot assays

For *in vitro* GST pull-down, the fragments of TYLCV or PaLCuCNV Rep gene and whitefly PCNA were amplified and cloned into pGEX-6p-1 and pET32a for fusion with GST and His, respectively. Primers are listed in Dataset S6. All recombinant proteins were expressed in *Escherichia coli* strain BL21 and purified. TYLCV or PaLCuCNV Rep-GST was bound to glutathione Sepharose beads (GE Healthcare, 17-5132-01) for 3 h at 4°C, the mixtures were centrifuged for 5 min at 100 × *g*, and the supernatants were discarded. His-tagged PCNA was added to the beads and incubated for 2 h at 4°C. After being centrifuged and washed five times with PBS, the bead-bound proteins were eluted by boiling in PAGE buffer for 5 min, and the proteins were separated by SDS/PAGE gel electrophoresis. The presence of target proteins was verified by immunoblotting with an anti-His monoclonal antibody (Abcam, ab213204) and an anti-GST monoclonal antibody (Abcam, ab92). An expression product from pET32a vector was used as a negative control.

For experiments with GST pull-down from insects, recombinant TYLCV or PaLCuCNV Rep-GST was bound to glutathione sepharose beads. Total proteins extracted from non-viruliferous whiteflies using cell lysis buffer (20 mM Tris·HCl, pH 7.5, 150 mM NaCl, 1% Triton X-100, 20 mM β-glycerophosphate, 10 mM NaF, 1 mM PMSF, 1 mM sodium orthovanadate, 10 mg/mL leupeptin, 2 mg/mL aprotinin, 1 mM EDTA) were added to the beads and incubated for 2 h at 4°C. After being centrifuged and washed five times with PBS, the bead-bound proteins were eluted by boiling in PAGE buffer for 5 min, and the presence of target proteins were verified by immunoblotting with an anti-GST monoclonal antibody and an anti-PCNA monoclonal antibody (Abcam, ab29). Expression product from pGEX-6p-1 vector was used as a negative control.

For protein detection from insects, whitefly protein extracts were separated by sodium dodecyl sulfate-polyacrylamide gel electrophoresis and transferred to polyvinylidine difluoride membranes. The membranes were blocked with 5% nonfat milk in phosphate-buffered saline (PBS; Sangon Biotech,

SB0627) with 0.1% Tween 20 (BBI Life Sciences, 9005-64-5) and then incubated with anti-CP, anti-PCNA or anti-actin (E021020-02, EarthOx) antibodies. After incubation with secondary antibody, signals were visualized with the ECL plus detection system (Bio-Rad, 170-5060).

dsRNA preparation

dsRNA specific to PCNA (whitefly genome database gene ID Bta06715), DNA PolδS2 (whitefly genome database gene ID Bta13584) and DNA PolδS3 (whitefly genome database gene ID Bta01628) of *B. tabaci* or GFP was synthesized using the T7 High Yield RNA Transcription kit (Vazyme, TR101), following the manufacturer's instructions. Briefly, the DNA template for dsRNA synthesis was amplified with primers containing the T7 RNA polymerase promoter at both ends (*SI Appendix*, Dataset S6), and the purified DNA template was then used to generate dsRNAs. Subsequently, the synthesized dsRNA was purified via phenol-chloroform precipitation and resuspended in nuclease-free water, and the concentration of dsRNA was quantified with a NanoDrop 2000 (Thermo Fisher Scientific). Finally, the quality and size of the dsRNAs were further verified via electrophoresis in a 2% agarose gel.

The effect of aphidicolin treatment on virus replication

Newly emerged whiteflies were first fed with 5 μM aphidicolin (dissolved in DMSO) and mixed with 15% sucrose in the feeding chamber. Approximately 100 whiteflies were released into each feeding chamber. The chamber was incubated in an insect-rearing room for 48 h. Whiteflies fed with 15% sucrose and DMSO were used as control groups. Next, the whiteflies were given a 6-h AAP on virus-infected plants and transferred to cotton. Whiteflies were collected at 0, 1, 2 and 4 days after the transfer and used for viral load quantification. For each time point, 10 female whiteflies were used as one sample, and three replicates were examined.

Image acquisition and processing

Fluorescence images were acquired using a Zeiss LSM 710 confocal microscope. Nuclear DNA was visualized using a 405-nm laser for excitation; the Dylight 488-labeled antibody and Dylight 549-labeled antibody were visualized using a 488-nm laser and a 561-nm laser for excitation, respectively. Maximum intensity projection images were generated using the ZEN 2012 digital imaging system. For fluorescence density quantification, a single in-focus plane was acquired. The corrected total cell fluorescence (CTCF) = integrated density - (area of selected cell × mean fluorescence of background readings), was measured with ImageJ (v1.50i, NIH) (8).

Statistical analysis

Data were presented as mean ± SEM of three independent biological replicates, unless otherwise noted. The independent-sample t-test was performed to compare two means, and one-way ANOVA followed by least significant difference test was applied for multiple comparisons. Given the type of samples, differences in scatter chart were analyzed by the non-parametric Mann-Whitney U test. All analyses were performed using SPSS (version 13) software.

Fig. S1.

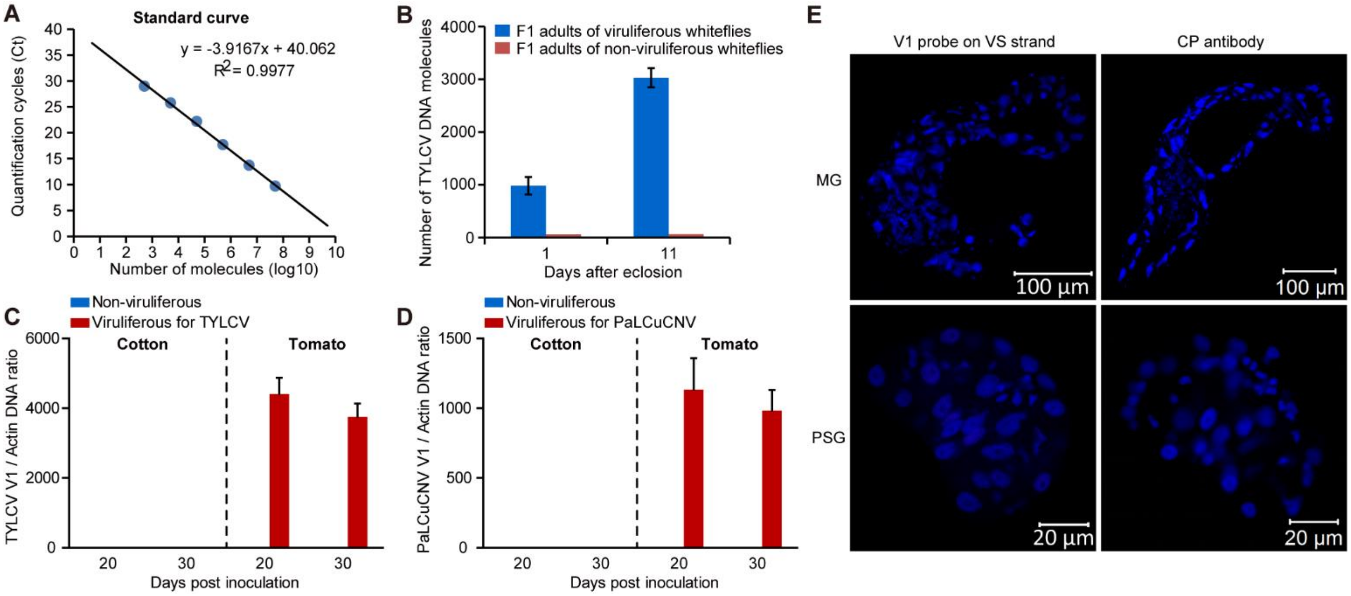

Fig. S1. Absolute quantification of TYLCV DNA molecules in F1 adults of MEAM1 whiteflies, virus transmission to plants by whiteflies and localization of TYLCV VS DNA and CP in non-viruliferous whiteflies. (*A*) Standard curve for absolute quantification of TYLCV DNA. Dilution series containing between 5×10^2 and 5×10^7 copies of circular dsDNA plasmids carrying a full-length copy of the viral genome were used as quantification standards. Data represent the average of three independent qPCR replicates. Equation used for regression plot calculation is indicated. (*B*) Absolute quantification of TYLCV DNA molecules in F1 adults of viruliferous and non-viruliferous whiteflies. Mean ± SEM of three independent experiments. (*C* and *D*) Transmission of TYLCV (*C*) or PaLCuCNV (*D*) to cotton and tomato plants by whiteflies. At 20 and 30 days post inoculation, newly emerged leaves were collected and used for virus DNA detection by qPCR. The *β-actin* gene of cotton or tomato was used as an internal control. (*E*) Localization of TYLCV VS DNA and CP in MG and PSG of non-viruliferous whiteflies. For TYLCV VS DNA localization, MG and PSG were hybridized with a Cy3-labeled VS strand-specific probe (V1 probe, red). TYLCV CP was detected by use of a mouse anti-CP monoclonal antibody and goat anti-mouse IgG labeled with Dylight 488 (green) secondary antibody. Cell nucleus was stained with DAPI (blue). For each tissue, 20 samples were analyzed and similar trend was observed.

Fig. S2.

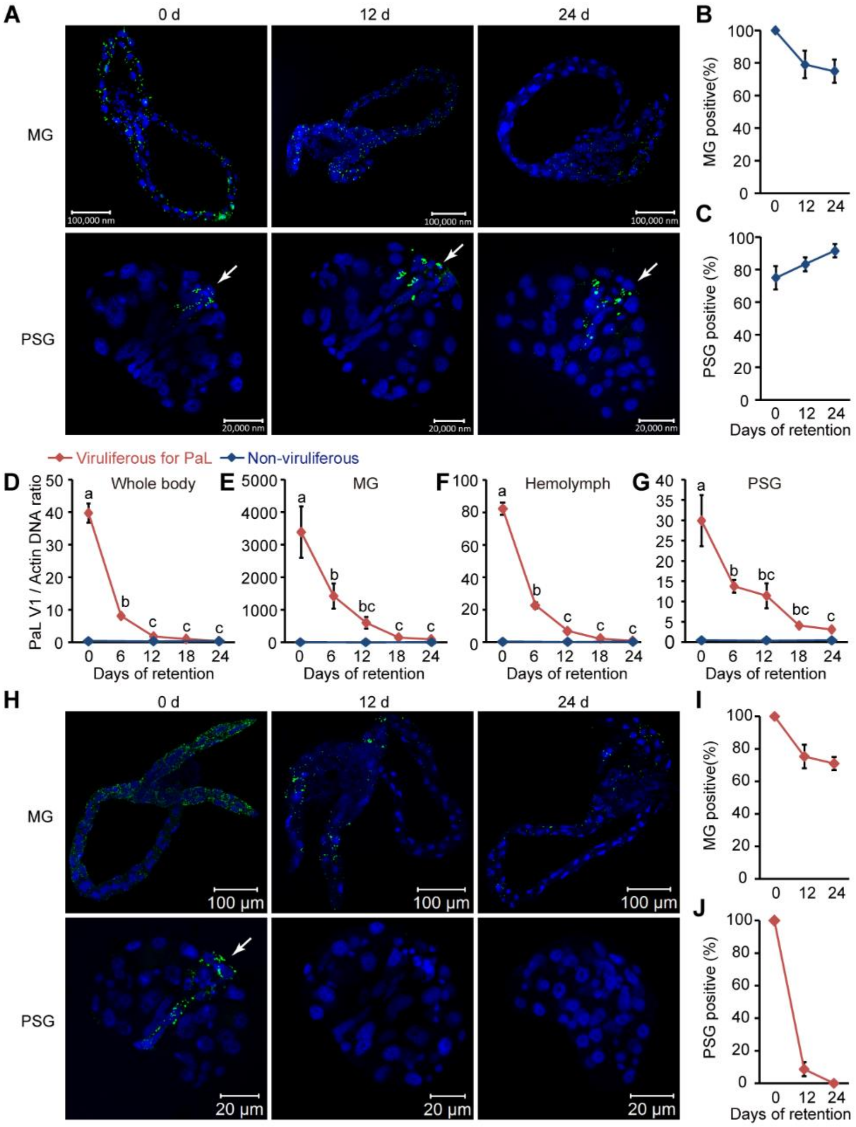

Fig. S2. Dynamics of TYLCV and PaLCuCNV in MEAM1 whiteflies during long-term retention. (*A*) Localization of TYLCV in MG and PSG of whiteflies after different times of retention. (*B* and *C*) The proportion of TYLCV-positive MG (*B*) and PSG (*C*) at each time point. MG (n = 24) and PSG (n = 24). (*D*-*G*) Relative concentration of PaLCuCNV (PaL) DNA in the whole body (*D*), MG (*E*), hemolymph (*F*) and PSG (*G*) of MEAM1 whiteflies. Total DNA was extracted from the whole body, MG, hemolymph and PSG for assay by qPCR. Mean ± SEM of three independent experiments. $P < 0.05$ (one-way ANOVA, LSD test). (*H*) Localization of PaLCuCNV in MG and PSG of whiteflies after different times of retention. (*I* and *J*) The proportion of PaLCuCNV-positive MG (*I*) and PSG (*J*) at each time point. MG (n = 24) and PSG (n = 24). (*A* and *H*) TYLCV and PaLCuCNV CP was detected using a mouse anti-CP monoclonal antibody and goat anti-mouse IgG labeled with Dylight 488 (green) secondary antibody. Cell nucleus was stained with DAPI (blue). The white arrow indicates of the virus signal in the PSG. (*B*, *C*, *I* and *J*) Mean ± SEM of three independent experiments.

Fig. S3.

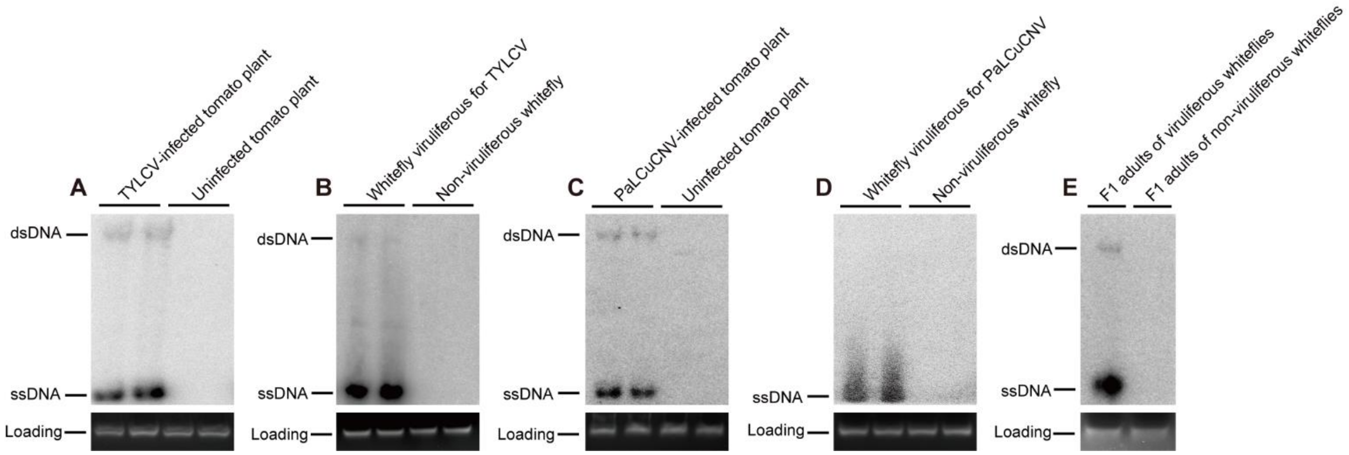

Fig. S3. Southern blot hybridization of TYLCV and PaLCuCNV DNA in plants and whiteflies. (A) Total DNA from TYLCV-infected or uninfected tomato plant was used for DNA gel blotting. 60 µg DNA was added for each lane. (B) Total DNA from whiteflies viruliferous for TYLCV or non-viruliferous whiteflies was used for DNA gel blotting. 200 µg DNA was added for each lane. (C) Total DNA from PaLCuCNV-infected or uninfected tomato plant was used for DNA gel blotting. 60 µg DNA was added for each lane. (D) Total DNA from whiteflies viruliferous for PaLCuCNV or non-viruliferous whiteflies was used for DNA gel blotting. 250 µg DNA was added for each lane. (E) Total DNA from first generation (F1) adults of whiteflies viruliferous for TYLCV or non-viruliferous whiteflies was used for DNA gel blotting. 200 µg DNA was added for each lane. Ethidium bromide-stained genomic DNA served as the loading control. dsDNA, double-stranded DNA. ssDNA, single-stranded DNA.

Fig. S4.

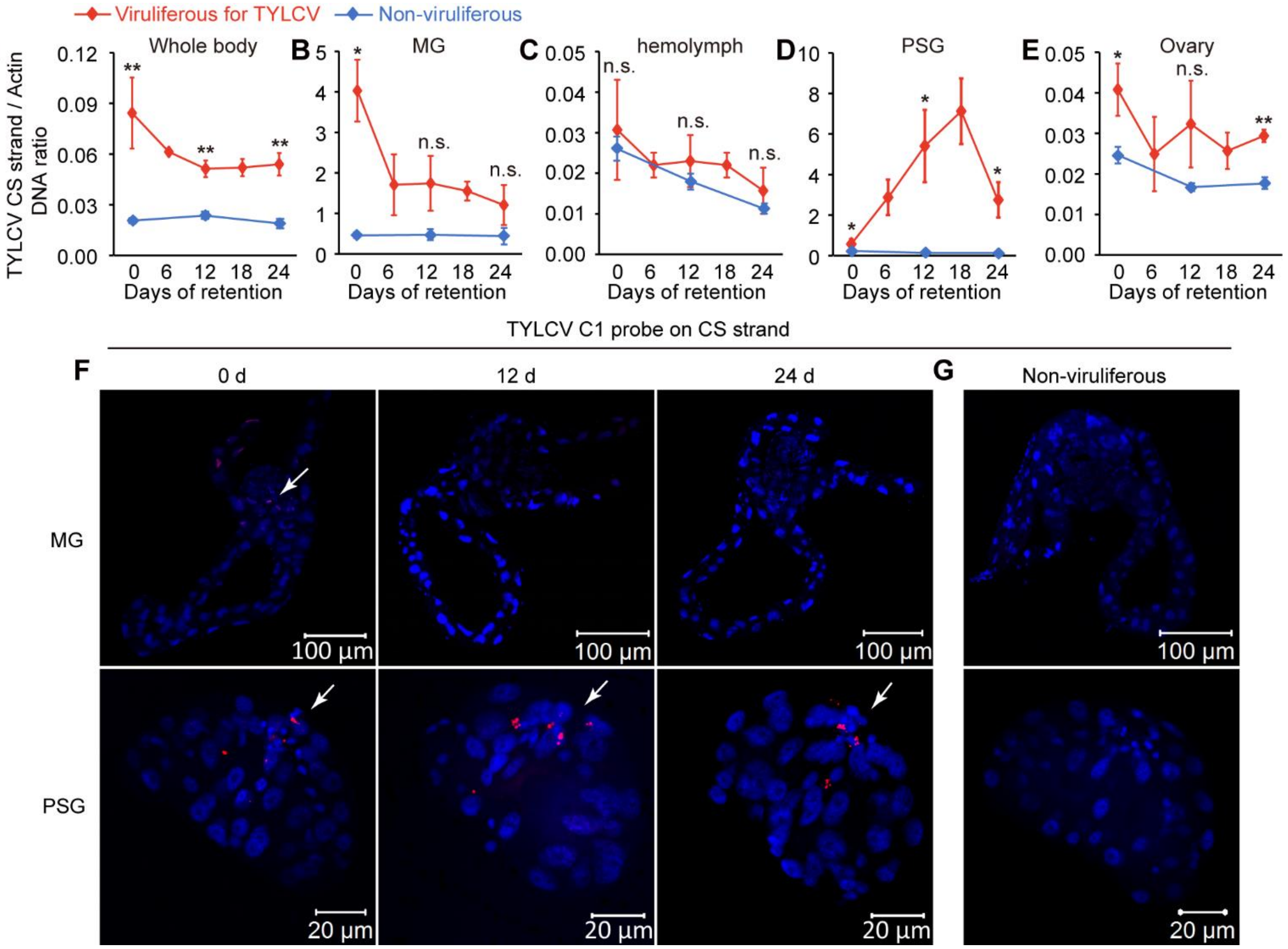

Fig. S4. Dynamics of the replicative complementary-sense (CS) DNA strand of TYLCV in MEAM1 whiteflies during long-term retention. (*A*-*E*) Relative concentration of TYLCV CS DNA strand in the whole body (*A*), MG (*B*), hemolymph (*C*), PSG (*D*) and ovary (*E*) of whiteflies. Total DNA was extracted from the whole body, MG, hemolymph, PSG and ovary and used in a two-step anchored qPCR for the specific quantification of the CS DNA strand of TYLCV. Mean ± SEM of three independent experiments. n.s., not significant, $^{*}P < 0.05$, $^{**}P < 0.01$ (independent-sample t-test). (*F*) Localization of TYLCV CS DNA in MG and PSG of viruliferous whiteflies after different times of retention. For each time point, 20 samples were analyzed and similar trend was observed. The white arrow indicates of the signal of TYLCV CS DNA. (*G*) Localization of TYLCV CS DNA in MG and PSG of non-viruliferous whiteflies. (*F* and *G*) MG and PSG were hybridized with a Cy3-labeled TYLCV CS strand-specific probe (C1 probe, red). Cell nucleus was stained with DAPI (blue).

Fig. S5.

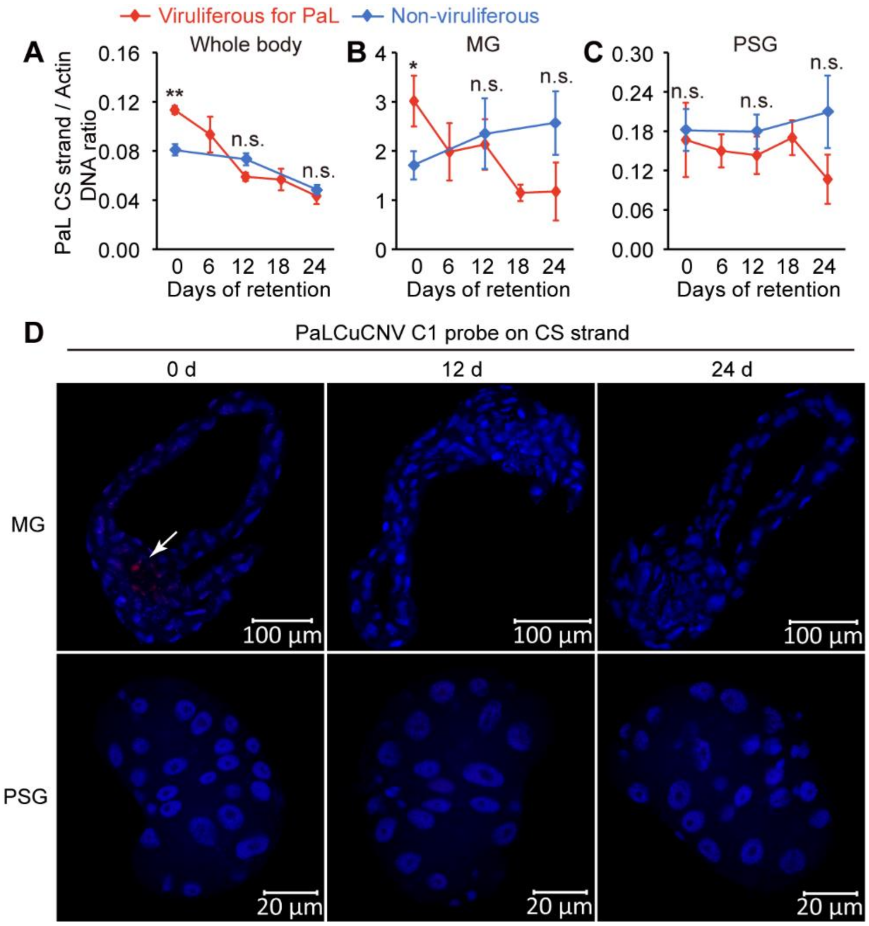

Fig. S5. Dynamics of the replicative complementary-sense (CS) DNA strand of PaLCuCNV in MEAM1 whiteflies during long-term retention. (*A*-*C*) Relative concentration of PaLCuCNV (PaL) CS DNA strand in the whole body (*A*), MG (*B*) and PSG (*C*) of whiteflies. Total DNA was extracted from the whole body, MG and PSG and used in a two-step anchored qPCR for the specific quantification of the CS DNA strand of PaL. Mean ± SEM of three independent experiments. n.s., not significant, $^{*}P < 0.05$, $^{**}P < 0.01$ (independent-sample t-test). (*D*) Localization of PaLCuCNV CS DNA in MG and PSG of viruliferous whiteflies after different times of retention. MG and PSG were hybridized with a Cy3-labeled PaLCuCNV CS strand-specific probe (C1 probe, red). The white arrow indicates of the signal of PaLCuCNV CS DNA. For each time point, 20 samples were analyzed and similar trend was observed.

Fig. S6.

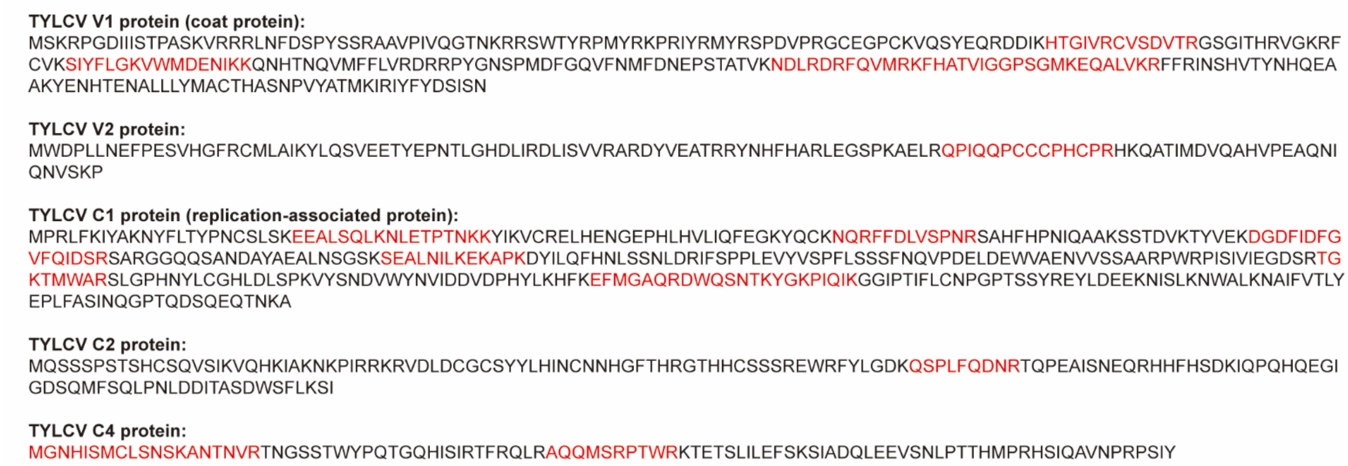

Fig. S6. Mapping of TYLCV-encoded peptides identified by mass spectrometry onto the primary amino acid sequences. Peptides identified from PSG of whiteflies viruliferous for TYLCV at 18 days of retention were indicated by red color.

Fig. S7.

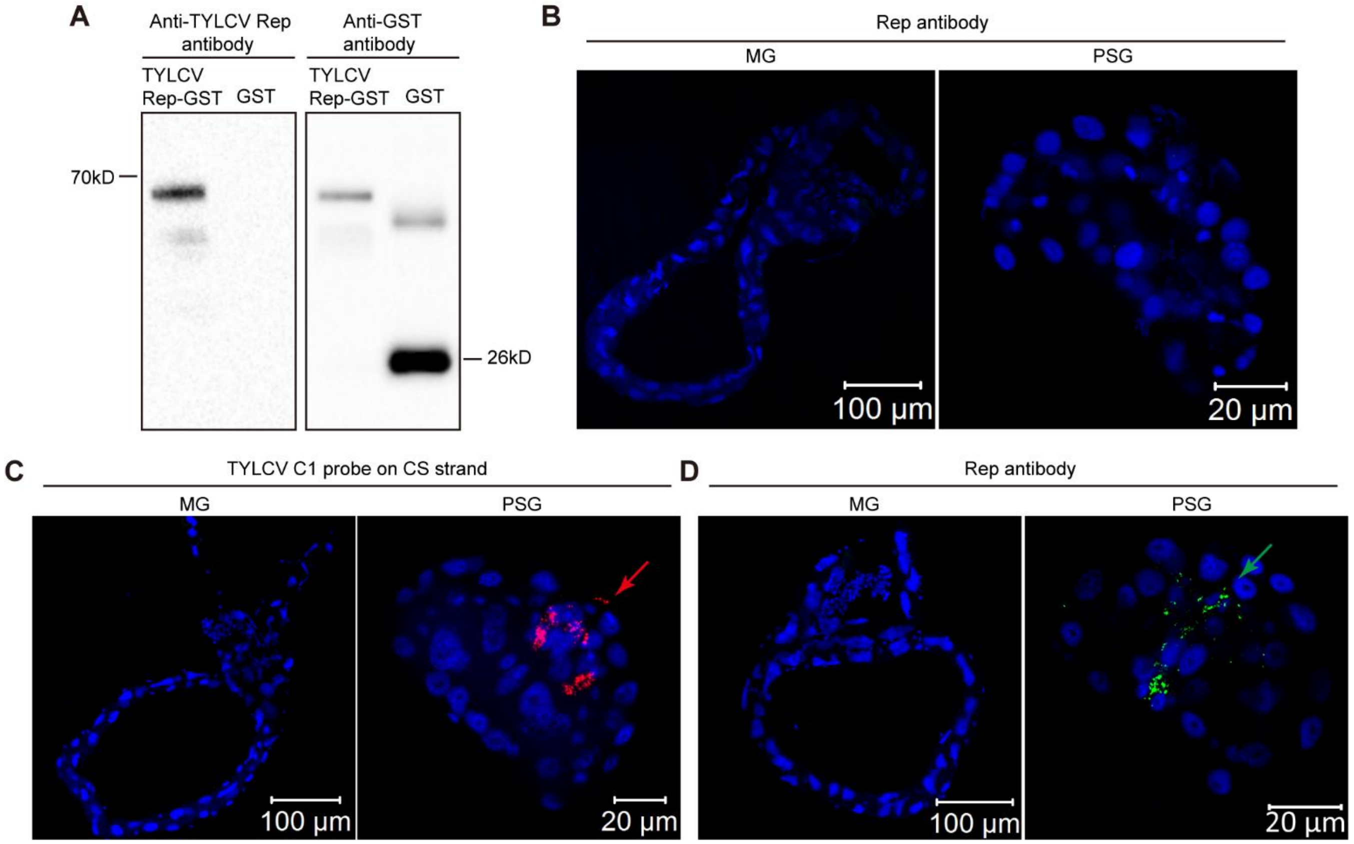

Fig. S7. Localization of the replicative CS DNA strand and Rep of TYLCV in MG and PSG of non-viruliferous MEAM1 whiteflies and adult offspring derived from viruliferous MEAM1 whiteflies. (*A*) Immunoblot of GST-fused TYLCV Rep using an anti-TYLCV Rep antibody (left panel) or anti-GST antibody (right panel). (*B*) Localization of TYLCV Rep in MG and PSG of non-viruliferous MEAM1 whiteflies. (*C* and *D*) Localization of the CS DNA (*C*) and Rep (*D*) of TYLCV in MG and PSG of 11 days after eclosion adult offspring derived from viruliferous MEAM1 whiteflies. For TYLCV CS DNA strand localization, MG and PSG were hybridized with a Cy3-labeled TYLCV CS strand-specific probe (C1 probe, red). Cell nucleus was stained with DAPI (blue). The red arrow indicates of the signal of TYLCV CS DNA in the PSG. The green arrow indicates of the signal of TYLCV Rep in the PSG. (*B* and *D*) Rep was detected by use of a rabbit anti-Rep polyclonal antibody and goat anti-rabbit IgG labeled with Dylight 488 (green) secondary antibody. For each tissue, 20 samples were analyzed and similar trend was observed.

Fig. S8.

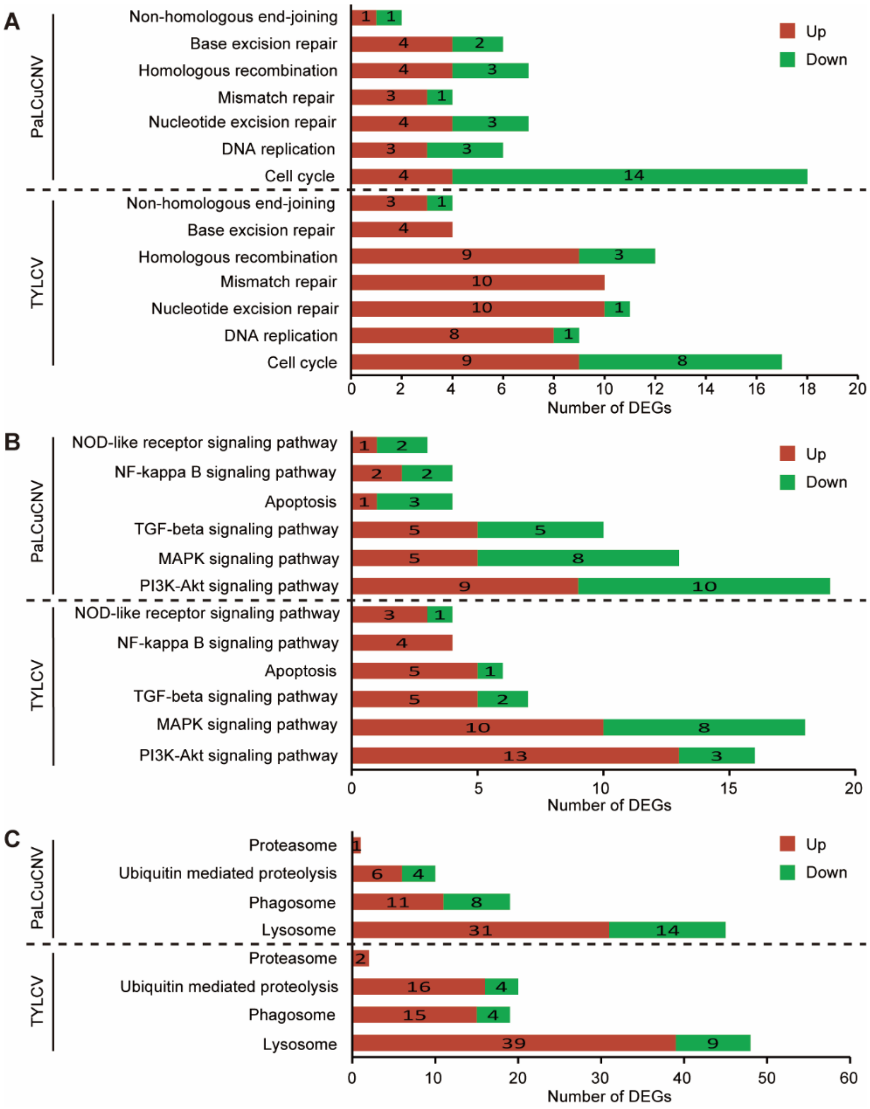

Fig. S8. Differentially expressed genes (DEGs) in PSG of viruliferous whiteflies. (*A*) Number of DEGs related to cell cycle, DNA replication and DNA repair. (*B* and *C*) Number of DEGs related to signal transduction pathway (*B*) and immune response (*C*). Genes contributing to each functional class are listed in Dataset S3 and Dataset S4.

Fig. S9.

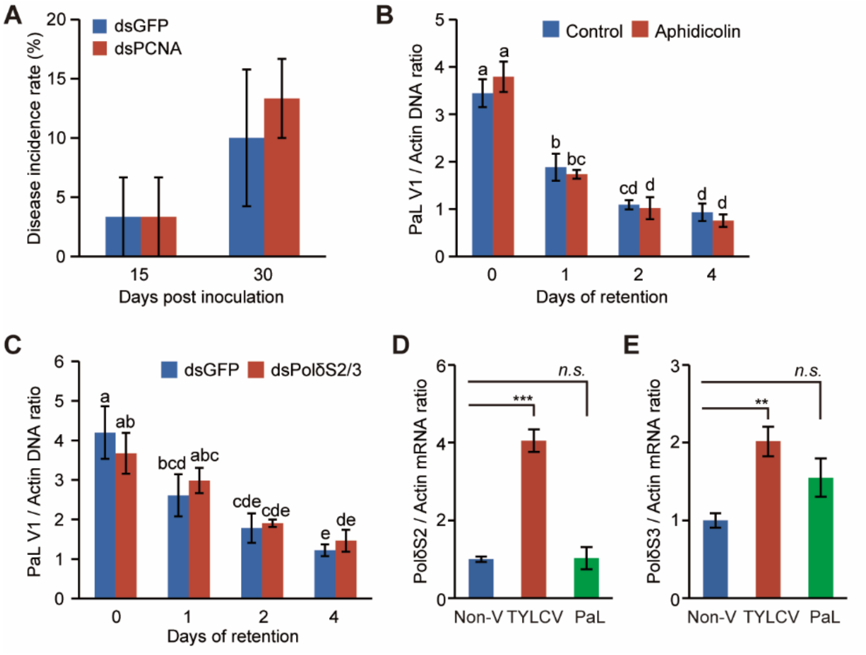

Fig. S9. The role of whitefly PCNA and DNA polymerase δ in PaLCuCNV transmission and accumulation. (*A*) The disease incidence rate of the tomato plants with PaLCuCNV fed upon by dsPCNA/dsGFP-treated whiteflies. (*B* and *C*) The effect of aphidicolin (*B*) or dsPolδS2/3 (*C*) treatment on PaLCuCNV (PaL) accumulation in whiteflies. Mean ± SEM of three independent experiments. $P < 0.05$ (one-way ANOVA, LSD test). (*D* and *E*) Relative expression levels of PolδS2 (*D*) and PolδS3 (*E*) in non-viruliferous (Non-V) whiteflies and whiteflies viruliferous for TYLCV or PaLCuCNV (PaL). Mean ± SEM of three independent experiments. *n.s.*, not significant, $^{**}P < 0.01$, $^{***}P < 0.001$ (independent-sample t-test).

Fig. S10.

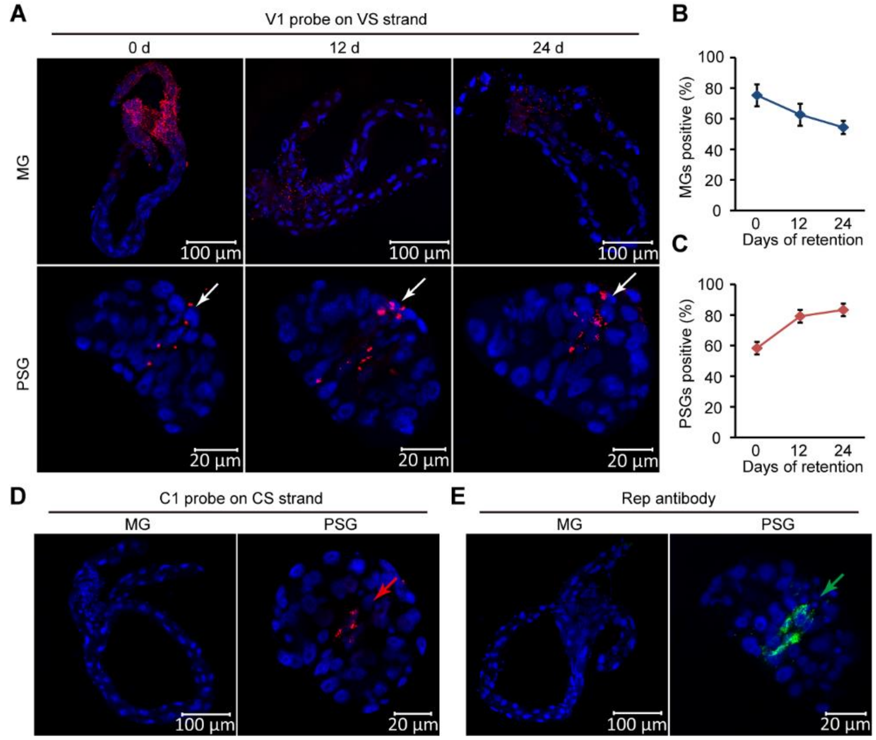

Fig. S10. Replication of TYLCV in PSG of MED whiteflies. (*A*) Localization of TYLCV VS DNA in MG and PSG of MED whiteflies after different time of retention. MG and PSG were hybridized with a Cy3-labeled TYLCV VS strand-specific probe (V1 probe, red). The white arrow indicates of the signal of TYLCV VS DNA in the PSG. (*B* and *C*) The proportion of TYLCV-positive MG (*B*) and PSG (*C*) at each time point. MG (n = 24) and PSG (n = 24). Mean ± SEM of three independent experiments. (*D* and *E*) Localization of TYLCV CS DNA strand (*D*) and Rep (*E*) in MG and PSG of MED whiteflies after 24 days of retention. For *D*, MG and PSG were hybridized with a Cy3-labeled TYLCV CS DNA strand-specific probe (C1 probe, red). For *E*, Rep was detected by use of a rabbit anti-Rep polyclonal antibody and goat anti-rabbit IgG labeled with Dylight 488 (green) secondary antibody. Cell nucleus was stained with DAPI (blue). The red arrow indicates of the signal of TYLCV CS DNA in the PSG. The green arrow indicates of the signal of TYLCV Rep in the PSG. For each tissue, 20 samples were analyzed and similar trend was observed.

Fig. S11.

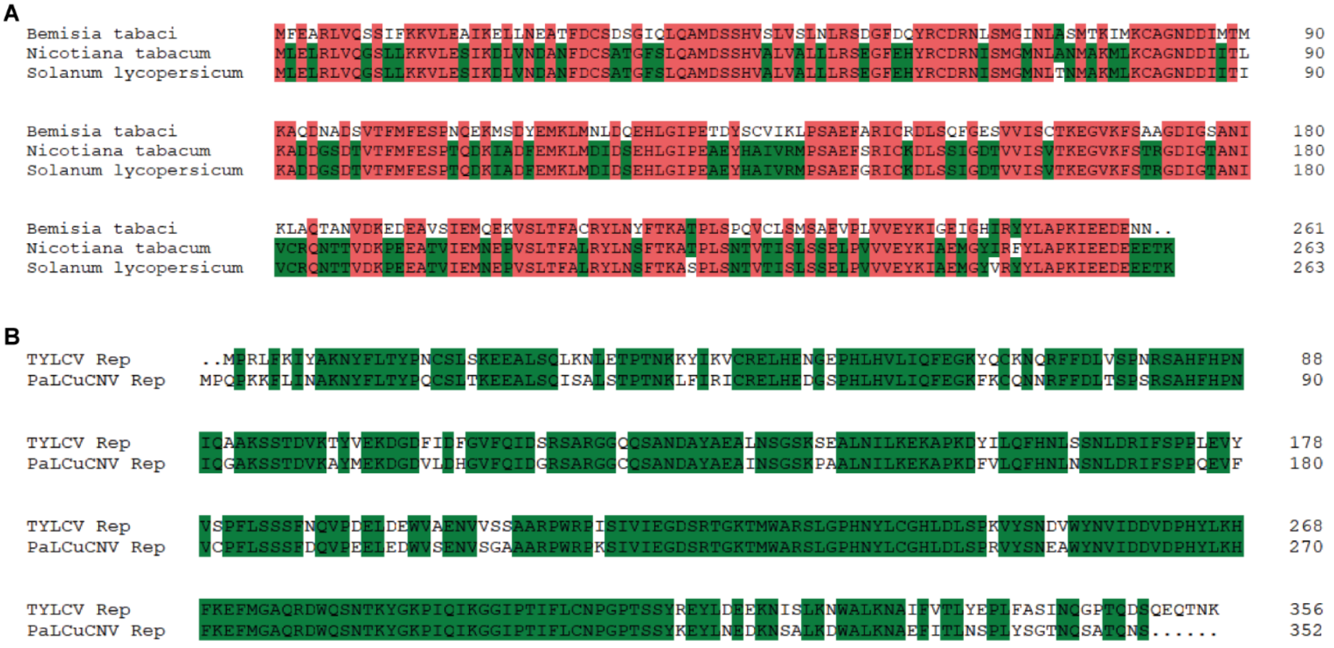

Fig. S11. Comparison of the amino acid sequences of host PCNA and viral Rep. (*A*) Comparison of the amino acid sequences of *Bemisia tabaci* PCNA (XP_018909891.1), *Nicotiana tabacum* PCNA (BAA76349.1) and *Solanum lycopersicum* PCNA (CAD56690.1). (*B*) Comparison of the amino acid sequences of TYLCV Rep (CAK54965.1) and PaLCuCNV Rep (CAX53751.1). Alignments were done by DNAMAN (6.0.3.93). Identical amino acids between two sequences are shown in black letters on a green background and identical amino acids between three sequences are shown in black letters on a red background.

Table S1. Localization of TYLCV virion-sense (VS) DNA strand and coat protein (CP) in midguts (MG) and primary salivary glands (PSG) of first generation (F1) adults of viruliferous MEAM1 whiteflies.

F1 adults developmental stage expressed as days after eclosion	Whitefly tissue	No. of samples with fluorescent signals/no. of samples observed	
		TYLCV VS DNA strand*	TYLCV CP†
1	MG	0/20	0/20
	PSG	14/20	12/20
11	MG	0/20	0/20
	PSG	16/20	15/20
31	MG	0/20	0/20
	PSG	13/20	11/20

*TYLCV VS DNA strand was hybridized with a Cy3-labeled VS strand-specific probe (V1 probe).

†TYLCV CP was detected by use of a mouse anti-CP monoclonal antibody and goat anti-mouse IgG labeled with Dylight 488 (green) secondary antibody.

Table S2. Localization of the replicative complementary-sense (CS) DNA strand of TYLCV or PaLCuCNV in viruliferous MEAM1 whiteflies after different times of retention.

		No. of samples with fluorescent signals/no. of samples observed	
Days of retention	Whitefly tissue	TYLCV CS DNA strand*	PaLCuCNV CS DNA strand*
0	MG	11/20	9/20
	PSG	15/20	0/20
12	MG	0/20	0/20
	PSG	17/20	0/20
24	MG	0/20	0/20
	PSG	15/20	0/20

*MG and PSG were hybridized with a Cy3-labeled CS strand-specific probe (C1 probe).

Table S3. Localization of TYLCV Rep in MG and PSG of whiteflies after different times of retention.

Days of retention	Whitefly tissue	No. observed	No. with signals*
0	MG	20	8
	PSG	20	11
18	MG	20	0
	PSG	20	18

*TYLCV Rep was detected by use of a rabbit anti-Rep polyclonal antibody and goat anti-rabbit IgG labeled with Dylight 488 (green) secondary antibody.

Dataset S1. Proteomic data of viral proteins in PSG of viruliferous whiteflies identified by mass spectrometry
Dataset S2. Differentially expressed genes in PSG from whiteflies viruliferous for TYLCV or PaLCuCNV
Dataset S3. TYLCV regulated genes and pathways in PSG
Dataset S4. PaLCuCNV regulated genes and pathways in PSG
Dataset S5. Genes verified by qRT-PCR
Dataset S6. The main primers and probes used in this study

SI References

1. De Barro PJ, Liu SS, Boykin LM, & Dinsdale AB (2011) *Bemisia tabaci*: A statement of species status. *Annu Rev Entomol* 56:1-19.
2. Rodríguez-Negrete EA, et al. (2014) A sensitive method for the quantification of virion-sense and complementary-sense DNA strands of circular single-stranded DNA viruses. *Sci Rep* 4:6438.
3. Czosnek H, *et al.* (1988) Detection of tomato yellow leaf curl virus in lysates of plants and insects by hybridization with a viral DNA probe. *Plant Dis* 72(11):949-951.
4. Wiśniewski JR (2016) Quantitative evaluation of filter aided sample preparation (FASP) and multienzyme digestion FASP protocols. *Anal Chem* 88(10):5438-5443.
5. Picelli S, *et al.* (2013) Smart-seq2 for sensitive full-length transcriptome profiling in single cells. *Nat Methods* 10:1096-1098.
6. Trapnell C, *et al.* (2012) Differential gene and transcript expression analysis of RNA-seq experiments with TopHat and Cufflinks. *Nat Protoc* 7:562-578.
7. Trapnell C, *et al.* (2010) Transcript assembly and quantification by RNA-Seq reveals unannotated transcripts and isoform switching during cell differentiation. *Nat Biotechnol* 28:511-515.
8. Mccloy RA, *et al.* (2014) Partial inhibition of Cdk1 in G2 phase overrides the SAC and decouples mitotic events. *Cell Cycle* 13:1400-1412.
